# Development of ^225^Ac-doped biocompatible nanoparticles for targeted alpha therapy

**DOI:** 10.1186/s12951-024-02520-6

**Published:** 2024-06-02

**Authors:** Miguel Toro-González, Ngozi Akingbesote, Amber Bible, Debjani Pal, Brian Sanders, Alexander S. Ivanov, Santa Jansone-Popova, Ilja Popovs, Paul Benny, Rachel Perry, Sandra Davern

**Affiliations:** 1 Isotope Science and Engineering Directorate, Oak Ridge National Laboratory, 1 Bethel Valley Road, Oak Ridge, TN 37830 USA; 2 Department of Cellular and Molecular Physiology, Yale University School of Medicine, New Haven, CT 06510 USA; 3 Biological and Environmental Systems Science Directorate, Oak Ridge National Laboratory, 1 Bethel Valley Road, Oak Ridge, TN 37830 USA; 4 Physical Sciences Directorate, Oak Ridge National Laboratory, 1 Bethel Valley Road, Oak Ridge, TN 37830 USA

**Keywords:** Actinium-225, Poly(lactic-*co*-glycolic acid), Nanoparticles, Therapy, Ligand

## Abstract

Targeted alpha therapy (TAT) relies on chemical affinity or active targeting using radioimmunoconjugates as strategies to deliver α-emitting radionuclides to cancerous tissue. These strategies can be affected by transmetalation of the parent radionuclide by competing ions in vivo and the bond-breaking recoil energy of decay daughters. The retention of α-emitting radionuclides and the dose delivered to cancer cells are influenced by these processes. Encapsulating α-emitting radionuclides within nanoparticles can help overcome many of these challenges. Poly(lactic-*co*-glycolic acid) (PLGA) nanoparticles are a biodegradable and biocompatible delivery platform that has been used for drug delivery. In this study, PLGA nanoparticles are utilized for encapsulation and retention of actinium-225 ([^225^Ac]Ac^3+^). Encapsulation of [^225^Ac]Ac^3+^ within PLGA nanoparticles (Z_ave_ = 155.3 nm) was achieved by adapting a double-emulsion solvent evaporation method. The encapsulation efficiency was affected by both the solvent conditions and the chelation of [^225^Ac]Ac^3+^. Chelation of [^225^Ac]Ac^3+^ to a lipophilic 2,9-bis-lactam-1,10-phenanthroline ligand ([^225^Ac]AcBLPhen) significantly decreased its release (< 2%) and that of its decay daughters (< 50%) from PLGA nanoparticles. PLGA nanoparticles encapsulating [^225^Ac]AcBLPhen significantly increased the delivery of [^225^Ac]Ac^3+^ to murine (E0771) and human (MCF-7 and MDA-MB-231) breast cancer cells with a concomitant increase in cell death over free [^225^Ac]Ac^3+^ in solution. These results demonstrate that PLGA nanoparticles have potential as radionuclide delivery platforms for TAT to advance precision radiotherapy for cancer. In addition, this technology offers an alternative use for ligands with poor aqueous solubility, low stability, or low affinity, allowing them to be repurposed for TAT by encapsulation within PLGA nanoparticles.

**Graphical Abstract:**

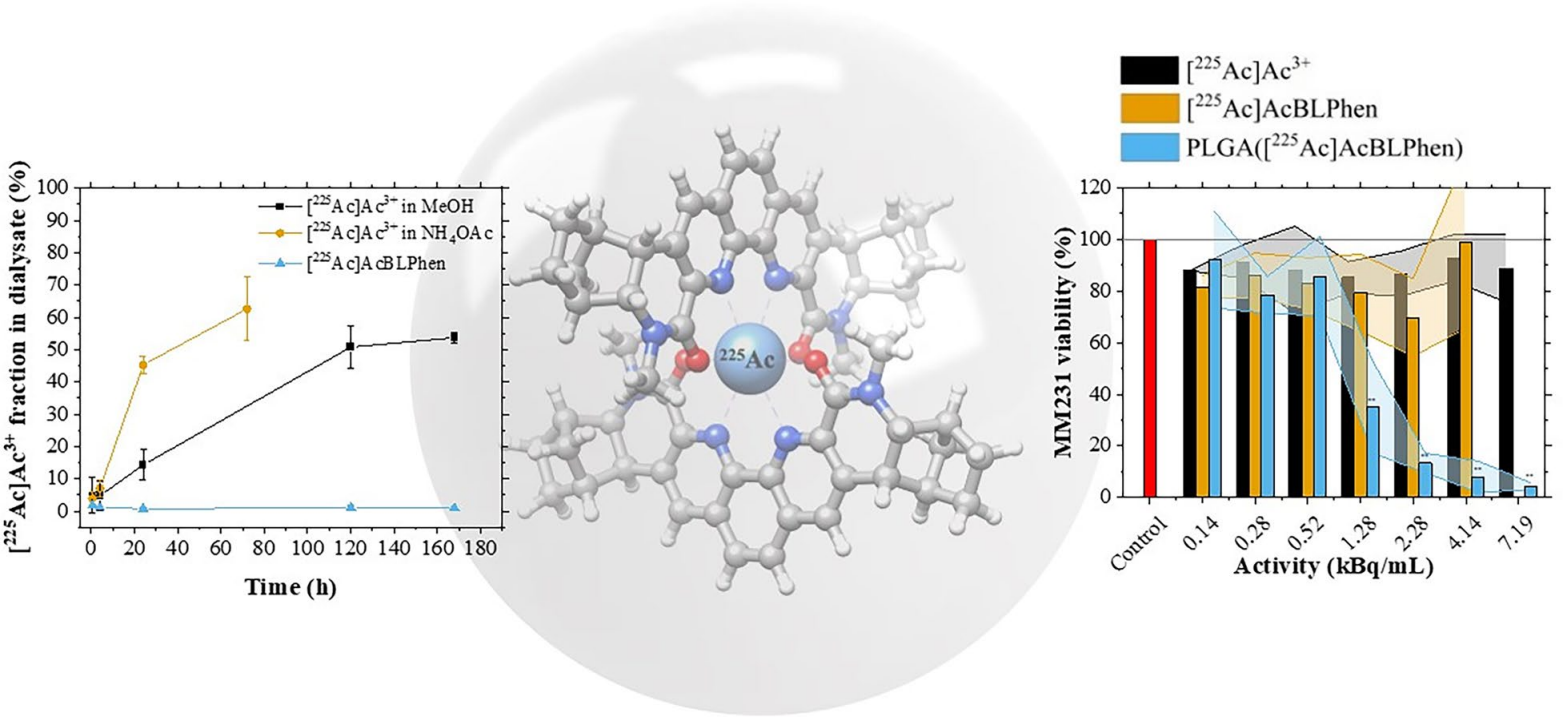

**Supplementary Information:**

The online version contains supplementary material available at 10.1186/s12951-024-02520-6.

## Introduction

Targeted alpha therapy (TAT) is a radiotherapy that uses α-emitting radionuclides for the precise treatment of cancer [1–3]. This precise treatment is attained by virtue of the high energy deposition of α-particles within a short range in tissue (< 100 µm) [2, 4]. These characteristics and the cytotoxicity of α-particles can lead to unwanted side effects if they relocate to healthy tissue [5–8]. To mitigate this issue, α-emitting radionuclides can be targeted to a specific tissue based on either chemical affinity or highly expressed tumor antigens [6, 9, 10]. Targeting by chemical affinity is the mechanism behind Xofigo^®^; a radiopharmaceutical composed of [^223^Ra]RaCl_2_ that targets hydroxyapatite in bone undergoing remodeling [10]. Because [^223^Ra]Ra^2+^ does not directly target cancer cells, it exerts its effects through indirect irradiation and bystander effects [11]. Direct targeting of cancer cells is facilitated using radioimmunoconjugates composed of an α-emitting radionuclide attached to a targeting vector usually using a bifunctional chelator [12]. Bifunctional chelators with high radiolabeling efficiency and high stability have been developed to minimize the accumulation of α-emitting radionuclides in healthy tissue [13–16]. Despite recent developments, preventing the relocation of decay daughters from target sites remains a challenge due to the bond-breaking recoil energy of α-particles [17]. To address this issue, different approaches have been proposed: local administration of α-emitting radionuclides, rapid uptake of the radioimmunoconjugate by the cell, or the encapsulation of α-emitting radionuclides within nanoparticles [18]. The latter is an attractive approach for delivering α-emitting radionuclides at therapeutic doses as long as the nanoparticles do not accumulate in healthy tissue [18].

Nanoparticles with various morphologies, structures, and characteristics have been developed for TAT using organic and inorganic compounds [19–21]. Organic nanoparticles have been radiolabeled with unchelated and chelated α-emitting radionuclides using ionophore-mediated mechanisms and passive entrapment, respectively. [22–27] Retention of the parent radionuclide has been demonstrated to be greater than 90%, whereas the retention of decay daughters is dependent on the nanoparticle size [22–26, 28]. Inorganic nanoparticles are radiolabeled with α-emitting radionuclides by either incorporation within the nanoparticle structure, adsorption, or surface attachment with a ligand [29–35]. Enhanced retention of decay daughters has been achieved by leveraging the high electron density, core–shell structure, and ion-exchange capacity of inorganic nanoparticles [31, 34]. These results support the potential of inorganic and organic nanoparticles as novel radiopharmaceuticals in preclinical and clinical studies. Translation of nanoparticles into the clinic has been hampered by technical challenges in large-scale production, a lack of understanding of their biological fate, and unknown long-term effects [36–38]. Some of these challenges can be overcome by using nanoparticles composed of biocompatible and biodegradable lipids and block-copolymers.

Poly(lactic-*co-*glycolic acid) (PLGA) is a diblock copolymer—approved by the U.S. Food and Drug Administration—extensively studied for drug delivery and tissue engineering applications [39, 40]. In radiotherapy, PLGA nanoparticles have been used for delivery of radionuclides that emit β-particles ([^177^Lu]Lu^3+^) and Auger electrons ([^111^In]In^3+^) [41–44]. Recently, Sporer et al. radiolabeled PLGA micelles with [^211^At]At^–^ and [^125^I]I^–^ using either a synthon-based approach or direct radiolabeling of the amphiphilic block copolymer [45]. In this work, PLGA nanoparticles were explored as a biodegradable and biocompatible delivery platform of [^225^Ac]Ac^3+^ for TAT. It is hypothesized that PLGA nanoparticles could increase the therapeutic potential of [^225^Ac]Ac^3+^ by increasing the retention of the parent and partially containing its decay daughters at the target site. It is also anticipated that PLGA’s status as an FDA-approved diblock copolymer will facilitate preclinical and clinical translation of PLGA nanoparticles encapsulating [^225^Ac]Ac^3+^ for therapeutic applications. Encapsulation of [^225^Ac]Ac^3+^ within PLGA nanoparticles was performed via active entrapment of the radionuclide during synthesis with a double-emulsion solvent evaporation method. This method was adapted to radiological applications by decreasing reagent volumes and using a cup horn sonicator. These changes were made to: (1) decrease the amount of radioactive waste generated during the optimization of synthesis parameters; and (2) contain the radioactive solutions within closed Eppendorf tubes during sonication. Different synthesis parameters were varied to enhance the encapsulation efficiency of [^225^Ac]Ac^3+^ within PLGA nanoparticles. A lipophilic 2,9-bis-lactam-1,10-phenanthroline ligand (BLPhen)—which has shown promise for lanthanide and actinide separation [46, 47]—was used to improve the encapsulation efficiency and retention of [^225^Ac]Ac^3+^. Encapsulation of [^225^Ac]Ac-macropa complexes was also evaluated due to the potential of this ligand to bind [^225^Ac]Ac^3+^ [48]. Retention of [^225^Ac]Ac^3+^ and its decay daughters, [^221^Fr]Fr^+^ and [^213^Bi]Bi^3+^, was assessed using dialysis against phosphate-buffered saline. The cytotoxicity of free [^225^Ac]Ac^3+^ and PLGA nanoparticles encapsulating [^225^Ac]AcBLPhen was evaluated in vitro using murine and human breast cancer cells. PLGA nanoparticles encapsulating [^225^Ac]AcBLPhen were significantly more cytotoxic to breast cancer cells than exposure to free ^225^Ac in solution. The enhanced cytotoxicity could be a combination of nanoparticle accumulation at the cell surface, nanoparticle uptake within cells, and the resultant retention of radionuclides near the cells. This study shows that PLGA nanoparticles offer an effective biocompatible delivery mechanism for α-emitting radionuclides that is facilitated by pre-chelation with a lipophilic ligand. PLGA nanoparticles could serve as a platform to repurpose ligands with poor aqueous solubility or low stability in TAT. These nanoparticles have the potential to also advance precision combination therapies by encapsulating a variety of therapeutic payloads, such as radionuclides and chemotherapeutic drugs [41, 42].

## Experimental methods

### Materials

The chemicals Resomer RG 503 H poly(D,L-lactide-co-glycolide) (lactide:glycolide 50:50, acid terminated, M_w_ 24,000–38,000), D-α-tocopherol polyethylene glycol 1000 succinate (vitamin E-TPGS, CAS: 9002–96-4), lanthanum chloride heptahydrate (LaCl_3_⋅7H_2_O, ACS reagent, CAS: 10,025–84-0), ethyl acetate (EtOAc, ≥ 99.5%, ACS reagent, CAS: 141–78-6), phosphate-buffered saline tablets (PBS, P4417), methanol (MeOH, ≥ 99.8%, ACS reagent, CAS: 67–56-1), ammonium acetate (NH_4_OAc, ≥ 98%, for molecular biology, CAS: 631–61-8), nitric acid (HNO_3_, 70%, ACS reagent, CAS: 7697–37-2), dimethyl sulfoxide (DMSO, ≥ 99.9%, ACS reagent, CAS: 67–68-5), dichloromethane (DCM, ≥ 99.9%, HPLC Plus, CAS: 75–09-2), and 3-(N-morpholino)propanesulfonic acid (MOPS, ≥ 99.5%, CAS: 1132–61-2) were obtained from Sigma Aldrich and used without further purification. Deionized water (DI H_2_O, 18.2 MΩ⋅cm) was obtained from a MilliporeSigma Milli-Q water purification system. Slide-A-Lyzer G2 dialysis cassettes (0.5 mL, 10,000 molecular weight cutoff) and the alamarBlue HS cell viability reagent were purchased from Thermo Scientific. The chemicals 1,2-Distearoyl-sn-Glycero-3-Phosphoethanolamine-N-azide (DSPE-N3, Cat# BP-26155) and cyanine5 red-emitting fluorophore with a dibenzocyclooctyne (Cy5-DBCO, Cat# D30F0) group were obtained from BROADPHARM and Lumiprobe, respectively.

### Poly(lactic-co-glycolic acid) nanoparticle synthesis

A double-emulsion solvent evaporation method was adapted for the synthesis of PLGA nanoparticles [49, 50]. The main changes include: (1) using a cup-horn sonicator (Q125-110, Qsonica) to generate emulsions; and (2) decreasing the reagent volumes to minimize radiological waste generation. For a typical synthesis, 100 µL of PLGA (40 mg/mL in EtOAc) was mixed with 10 µL of a polar solution (i.e., DI H_2_O, MeOH, or a mixture of both) in an Eppendorf tube. This mixture was sonicated for 30 s in a cup-horn sonicator (100% amplitude) to generate a water/oil emulsion. After sonication, 200 µL of vitamin E-TPGS (1 wt % in DI H_2_O) was added immediately to the Eppendorf tube and mixed using a vortex mixer. Three sonication cycles of 30 s (100% amplitude) with a 30 s rest time were used to generate a water/oil/water emulsion. The water/oil/water emulsion was added to a beaker containing 2.5 mL of vitamin E-TPGS (0.5 wt % in DI H_2_O). These suspensions were stirred at 500–700 rpm for at least 4 h to harden the nanoparticles. After hardening, PLGA nanoparticle suspensions were washed at least three times by centrifugation (15,000 rpm, 20 min, 8 ºC) followed by resuspension and sonication in 1 mL DI H_2_O. The parameters and steps just mentioned were the standard conditions employed for the synthesis of PLGA nanoparticles. For radionuclide retention and cell viability studies, water/oil/water emulsions were transferred into a dialysis cassette and dialyzed against PBS (90 mL) stirring constantly (120 rpm). Emulsions were dialyzed for multiple days to assess the radionuclide retention. For cell-based assays, emulsions were dialyzed for at least 4 h in PBS stirring constantly (120 rpm) before diluting them in cell media. The water in the cup-horn sonicator was kept cold by adding ice during the emulsification steps described above.

**Fig. 1:**
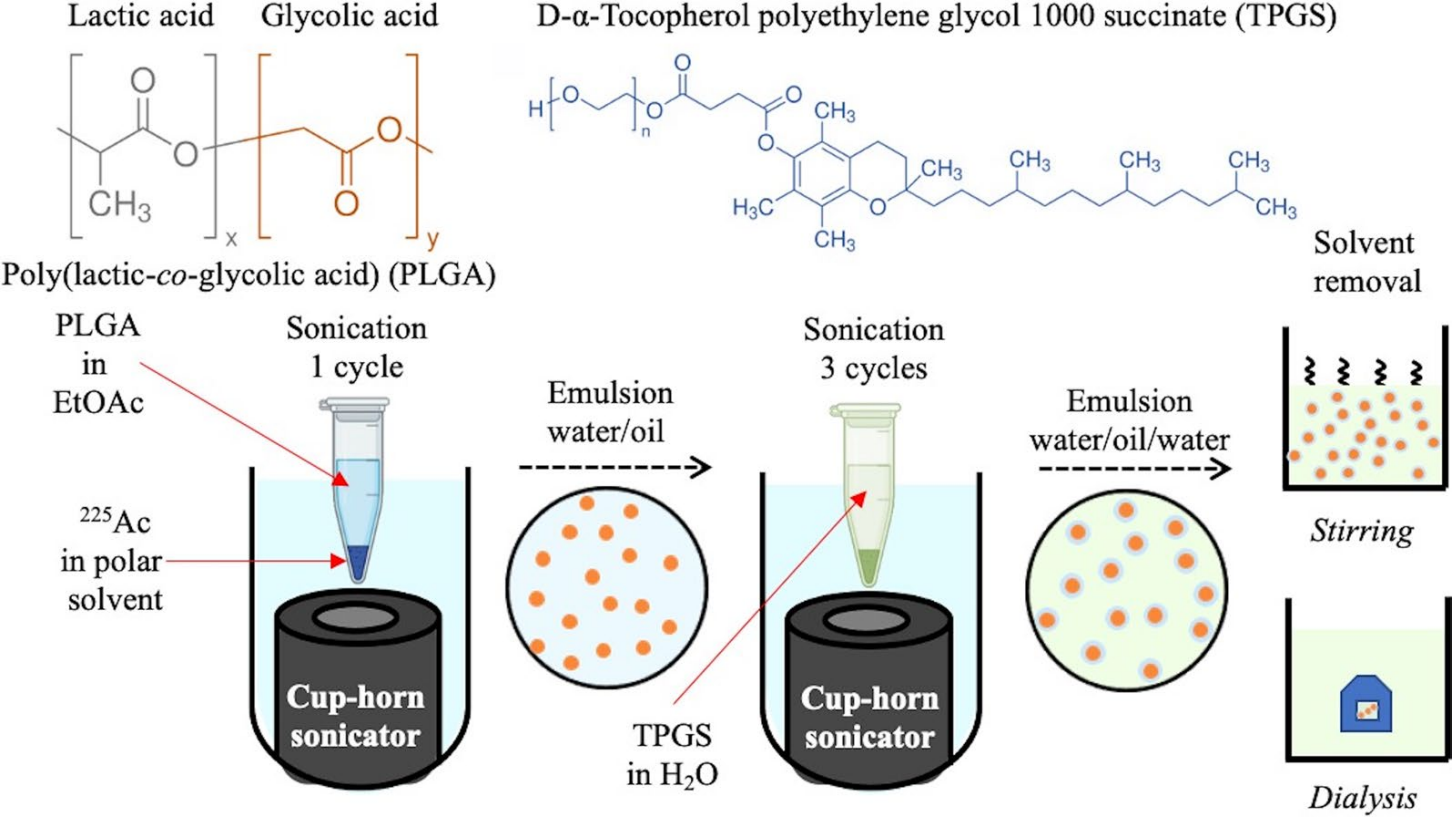
Controlled synthesis of PLGA nanoparticles with a cup-horn sonicator. Schematic representation of PLGA nanoparticles synthesized with a double-emulsion solvent evaporation method. Chemical structure of PLGA and TPGS are shown as a reference. The presence of methyl side groups in poly(lactic acid) [gray] makes it more hydrophobic than poly(glycolic acid) [orange]

A schematic representation of the PLGA nanoparticle synthesis method using double-emulsion solvent evaporation and a cup-horn sonicator is shown in Fig. 1. The influence of synthesis parameters on the size distribution of PLGA nanoparticles was assessed by varying PLGA and vitamin E-TPGS concentrations, PLGA payload, PLGA:TPGS volume ratios, sonication time and number of cycles, among other conditions.

Surrogate studies used LaCl_3_ in dilute HNO_3_ or in MOPS buffer as a payload during synthesis of PLGA nanoparticles. In these experiments, 10 µL of LaCl_3_ solution was mixed with 200 µL of PLGA solution (40 mg/mL in EtOAc) in an Eppendorf tube. The mixture was sonicated for 30 s and then 400 µL of vitamin E-TPGS (1 wt % in DI H_2_O) was added. Three sonication cycles of 30 s with a 30 s rest time were used to generate the water/oil/water emulsion. This emulsion was transferred into a beaker with 4 mL of vitamin E-TPGS (1 wt % in DI H_2_O) and stirred overnight at 700 rpm. PLGA nanoparticle suspensions were collected by centrifugation and subsequently washed with DI H_2_O. Both the PLGA nanoparticle suspension and the supernatant were assayed to determine the concentration of La and calculate its encapsulation efficiency within the nanoparticles. Copper-free click chemistry was used to prepare fluorescent PLGA nanoparticles by combining an azido phospholipid molecule and a cyanine5 red-emitting fluorophore with a dibenzocyclooctyne group [51, 52]. Briefly, 100 µL of PLGA (40 mg/mL), 12 µL DSPE-N3 (2.5 mg/mL), and 4 µL Cy5-DBCO (1 mg/mL) were mixed in an Eppendorf tube and sonicated for 30 s. The following steps for PLGA nanoparticle synthesis were performed as described above as the standard conditions.

### Characterization

Morphological characterization was performed by scanning electron microscopy (SEM) using a Carl Zeiss MERLIN VP-SEM with a secondary electron detector operating at 1 kV accelerating voltage. A PLGA nanoparticle suspension was diluted tenfold in DI H_2_O and then drop-casted onto a silicon wafer for SEM characterization. The size distribution of PLGA nanoparticle suspensions—diluted tenfold in DI H_2_O—was characterized using dynamic light scattering (DLS) in a Malvern Zetasizer Nano ZS. PLGA nanoparticle suspensions prepared with different conditions were characterized in duplicate to determine the mean hydrodynamic size and polydispersity index (PDI). The stability of PLGA nanoparticles was assessed over time after dilution in DI H_2_O, PBS, and cell media, respectively. Characterizations correspond to three runs with at least ten measurements each as defined in the DLS software. The DLS instrument was also used to measure the zeta potential of PLGA nanoparticles. Changes in the zeta potential after exposure to cell media were used to determine protein adsorption. PLGA nanoparticles were exposed to cell media, precipitated by centrifugation, and dispersed in DI H_2_O for zeta potential characterization. Inductively coupled plasma mass spectrometry (ICP-MS) was performed using a ThermoFisher Scientific iCAP-RQ quadrupole ICP-MS instrument to assess the concentration of La within PLGA nanoparticles. Sample preparation, quantification, and analysis were performed as described previously [49].

### Encapsulation and retention of radionuclides

Actinium-225 ([^225^Ac]Ac^3+^, t_1/2_ = 9.9 d) was obtained from a thorium generator ([^228^Th]Th^4+^, [^229^Th]Th^4+^, and [^232^Th]Th^4+^) at Oak Ridge National Laboratory after various chemical separation processes involving anion and cation exchange chromatography [53]. For encapsulation and retention experiments, an [^225^Ac]Ac^3+^ stock solution in 0.1 M HNO_3_ was mixed with NH_4_OAc (0.1 M, pH 6.0), MeOH, or a 5 mM lipophilic BLPhen ligand solution in MeOH. Details on the synthesis of the lipophilic BLPhen ligand can be found elsewhere [47]. Encapsulation of [^225^Ac]Ac-macropa was also explored because of the high stability of this complex [48]. Chelation of [^225^Ac]Ac^3^ with macropa [2.7 mM] was performed as reported by Thiele et al. [48] Chelation of [^225^Ac]Ac^3+^ by BLPhen and macropa was assessed using thin-layer chromatography with alumina-backed silica plates and sodium citrate (0.4 M in 10% MeOH) as stationary and mobile phases, respectively. Autoradiography of the plates was performed using a Cyclone Plus phosphor imager (PerkinElmer) and super-resolution phosphor screens.

Synthesis of PLGA nanoparticles encapsulating [^225^Ac]Ac^3+^ was performed using standard conditions. A 10 µL solution of ^225^Ac in NH_4_OAc, a MeOH/DI H_2_O mixture, or [^225^Ac]AcBLPhen in a MeOH/DI H_2_O mixture was used as the payload. The activity of [^225^Ac]Ac^3+^ in these solutions ranged between 3.7 kBq and 55.5 kBq (0.1 µCi and 1.5 µCi, respectively). Adjusting the [^225^Ac]Ac^3+^ activity resulted in various MeOH:DI H_2_O volume ratios (e.g., 95:5, 90:10, and 75:25) during PLGA nanoparticle synthesis. PLGA nanoparticle suspensions were washed three times with DI H_2_O to determine the encapsulation efficiency. After each centrifugation, the supernatant was carefully removed with a transfer pipette and stored in a 15 mL Falcon tube. To account for total radioactivity distribution, the PLGA nanoparticle suspension, the supernatant, and the Eppendorf tube used for sonication were assayed using γ-ray spectroscopy on a high purity germanium (HPGe) detector. The HPGe detector had a crystal active volume of ~ 100 cm^3^ and a beryllium window; and was coupled to a PC-based multichannel analyzer (Canberra Industries, Meriden, CT). Energy and efficiency calibrations were determined by γ-ray sources traceable to the National Institute of Standards and Technology. Radionuclide retention was assessed by quantifying the fraction of [^225^Ac]Ac^3+^, [^221^Fr]Fr^+^ (t_1/2_ = 4.5 min), and [^213^Bi]Bi^3+^ (t_1/2_ = 45.6 min) activity in the dialysate over time. The water/oil/water emulsion (~ 0.5 mL) was transferred into a dialysis cassette and dialyzed against PBS (90 mL) stirring constantly (120 rpm). Aliquots (5 mL) were taken from the dialysate at different time points (e.g., 0.5, 4, 24, 120, and 168 h) and assayed immediately on an HPGe detector to quantify the activity of [^221^Fr]Fr^+^ and [^213^Bi]Bi^3+^ [54, 55]. The same aliquot was counted the next day—decay daughters are now in equilibrium with [^225^Ac]Ac^3+^—to assess the fraction of [^225^Ac]Ac^3+^ in the dialysate [54, 55]. The equations used to determine the encapsulation efficiency and retention of radionuclides are presented in the supporting information. A 5 mL solution of fresh PBS was added to the beaker to maintain a constant dialysate volume.

### ***Quantum chemical calculations of [***^***225***^***Ac]AcBLPhen***

Electronic structure calculations of the [^225^Ac]AcBLPhen complex were performed employing density functional theory (DFT) with the Gaussian 16, Revision A.03 software package [56]. We utilized the hybrid B3LYP functional [57, 58] and the DFT-D3 approach of Grimme with zero damping [59] to account for van der Waals interactions. A standard 6–31 + G(d) basis set was used for main group elements and hydrogen for geometry optimizations. The *f*-element Ac was modeled using large-core (LC) relativistic effective core potential (RECP) and the associated (7s6p5d)/[4s3p3d] basis set [60]. Since LC RECP calculations include the 4*f* electrons in the core, they were performed on a pseudo singlet state configuration. Frequency calculations at the B3LYP-D3/LC/6–31 + G(d) level were performed to ensure real vibrational modes for the minimum ground state structures and to provide zero-point energies (ZPEs). ZPEs and thermal corrections (T = 298.15 K) were added to the total energy to obtain the Gibbs free energy. Thermal contributions to the gas-phase Gibbs energies were calculated using standard molecular thermodynamic approximations [61], except that vibrational frequencies lower than 60 cm^–1^ were raised to 60 cm^–1^, adopting a methodology introduced by Truhlar et al. [62] based on the so-called quasiharmonic approximation. Using the gas-phase geometries, implicit solvent corrections were obtained at 298.15 K with the SMD [63] solvation model as implemented in Gaussian 16. Complexation free energies in aqueous solution, Δ*G*_aq_, were calculated using the thermodynamic cycle approach described in our previous works on vanadium- and uranyl-containing complexes [64, 65]. Chemical bonding analysis was performed for the DFT-optimized structures using the natural bond orbital (NBO) methodology [66].

### Tissue culture

Cell lines E0771 murine breast carcinoma (CRL-3461™), MCF-7 human breast adenocarcinoma (HTB-22), and MDA-MB-231 human breast adenocarcinoma (HTB-26) were obtained from the American Type Culture Collection. Cells were seeded in a 75 cm^2^ tissue culture flask and cultured at 37 °C in humidified air containing 5% CO_2_. A 1:1 mixture of Dulbecco’s Modified Eagle’s Medium and Ham’s F12 medium, supplemented with 10% fetal calf serum, 2 mM L-glutamine, 100 I.U./mL penicillin, and 100 µg/µL streptomycin, was used to culture the three cell lines (DMEM/F12 complete media). Cells were passaged by trypsinization and seeded at ~ 3 × 10^3^ cells per well in a flat-bottom tissue culture–treated 96-well plate (Corning Costar). Cells were incubated for 48 h at 37 °C in humidified air containing 5% CO_2_, reaching 70% to 80% confluency before treatment.

Breast cancer cell lines were exposed for 24 h to different [^225^Ac]Ac^3+^ activities delivered as free [^225^Ac]Ac^3+^, [^225^Ac]AcBLPhen, and PLGA nanoparticles encapsulating [^225^Ac]AcBLPhen. Radioactive solutions were initially prepared in PBS and then serially diluted into DMEM/F12 complete media. The activities used in this work ranged between 0.14 ± 0.01 kBq/mL and 7.2 ± 1.6 kBq/mL (3.7 ± 0.2 nCi/mL and 194 ± 42 nCi/mL, respectively). This activity range was defined based on in vitro experiments with targeted [^225^Ac]Ac^3+^ and [^225^Ac]Ac^3+^ encapsulated within nanoparticles [30, 32, 67–70]. After 24 h of exposure to [^225^Ac]Ac^3+^, the radioactive media (200 µL/well) was removed and cells were washed three times with PBS. Metabolic activity of cells was used as a measure of viability after exposure to [^225^Ac]Ac^3+^. Cell viability was assessed 1 h and 72 h after exposure to [^225^Ac]Ac^3+^ using the alamarBlue HS cell viability reagent. Cell viability was calculated as percent viability relative to untreated cells, a negative control. AlamarBlue reagent was mixed with DMEM/F12 complete media (10 µL alamarBlue per 90 µL media) and then added to the 96-well plate (100 µL per well). Cells were incubated at 37 °C for 1 h before measuring the alamarBlue fluorescence (λ_exc_ = 560 nm and λ_emi_ = 590 nm) with a BioTek Cytation 1 cell imaging multimode reader (red filter block, λ_exc_ = 530 nm and λ_emi_ = 590 nm). After the fluorescence was recorded, the alamarBlue media was removed and replaced with fresh DMEM/F12 complete media, and cells were incubated at 37 °C, 5% CO_2_ in the humidified incubator. The viability assay was repeated 72 h after exposure to ^225^Ac following the steps described previously. To assess the influence of nanoparticle concentration on cell viability, cells were exposed for 24 h to similar [^225^Ac]Ac^3+^ activities delivered by PLGA nanoparticles prepared with different polymer concentrations (e.g., 10 mg/mL, 20 mg/mL, and 40 mg/mL). The PLGA nanoparticle concentration ranged between 16.7 µg/mL and 266.7 µg/mL. The viability assay for the different nanoparticle concentrations was performed as described previously 1 h post-exposure. To determine the effect of exposure time on cell viability, E0771 and MCF-7 cells were exposed to free [^225^Ac]Ac^3+^ in PBS and PLGA nanoparticles encapsulating [^225^Ac]AcBLPhen for different time points (e.g., 1 h, 3 h, 6 h, and 24 h). Cell viability was evaluated 24 h and 48 h after exposure to ^225^Ac.

Immunostaining for γ-H2AX was performed following the protocol established by Noubissi et al. [71] After exposure to [^225^Ac]Ac^3+^, cells were washed with PBS (× 3) and fixed using a 4% (v/v) paraformaldehyde solution in PBS for 10 min. After washing the cells with PBS (× 3), they were permeabilized with a 0.3% (v/v) Triton X-100 solution in PBS for 10 min. Following permeabilization, cells were washed with PBS (× 3) and blocked with a MAXblock blocking solution overnight at 4 ºC. After washing the cells with PBS (× 3), cells were incubated with the primary antibody for 1 h at 37 ºC followed by 30 min at room temperature with rocking. The primary antibody—Anti-phospho-Histone H2A.X (serine 139) clone JBW301 (MilliporeSigma) —was diluted to 2 µg/mL in MAX bind staining medium. Following this incubation, cells were washed with PBS (× 3) and incubated with the fluorescent-tagged secondary antibody. The secondary antibody—Anti-Mouse IgG (H + L), F(ab’)2 fragment CF 488A (Millipore Sigma)—was diluted to 1 µg/mL in MAX bind staining medium. Incubation with the secondary antibody was performed for 1 h at 37ºC. After incubation, cells were washed with PBS (× 3) and stained with Hoechst. Hoechst (10 mg/mL) was diluted in PBS (1:2000) and incubated for 5 min at room temperature. Cells were washed with PBS (× 3) and maintained in 100 µL of PBS for imaging and image acquisition. Images were acquired using a Biotek Cytation 1 cell imaging multi-mode reader (Biotek Instruments Inc.). The filter cubes used to image the cells were DAPI (Cat #1,225,100) and GFP (Cat #1,225,101). A spot counting macro in ImageJ was used for direct foci counting.

Interaction of fluorescent PLGA nanoparticles with E0771 and MDA-MB-231 cells was evaluated using confocal microscopy. Cells were seeded at ~ 3 × 10^3^ cells per well in a 6-well plate, incubated for 48 h at 37 °C in humidified air containing 5% CO_2_, and treated with 16.5 µg/mL and 66.4 µg /mL of fluorescent PLGA nanoparticles per well. Images were recorded after 2 h and 24 h of incubation with PLGA nanoparticles.

## Results

**Fig. 2:**
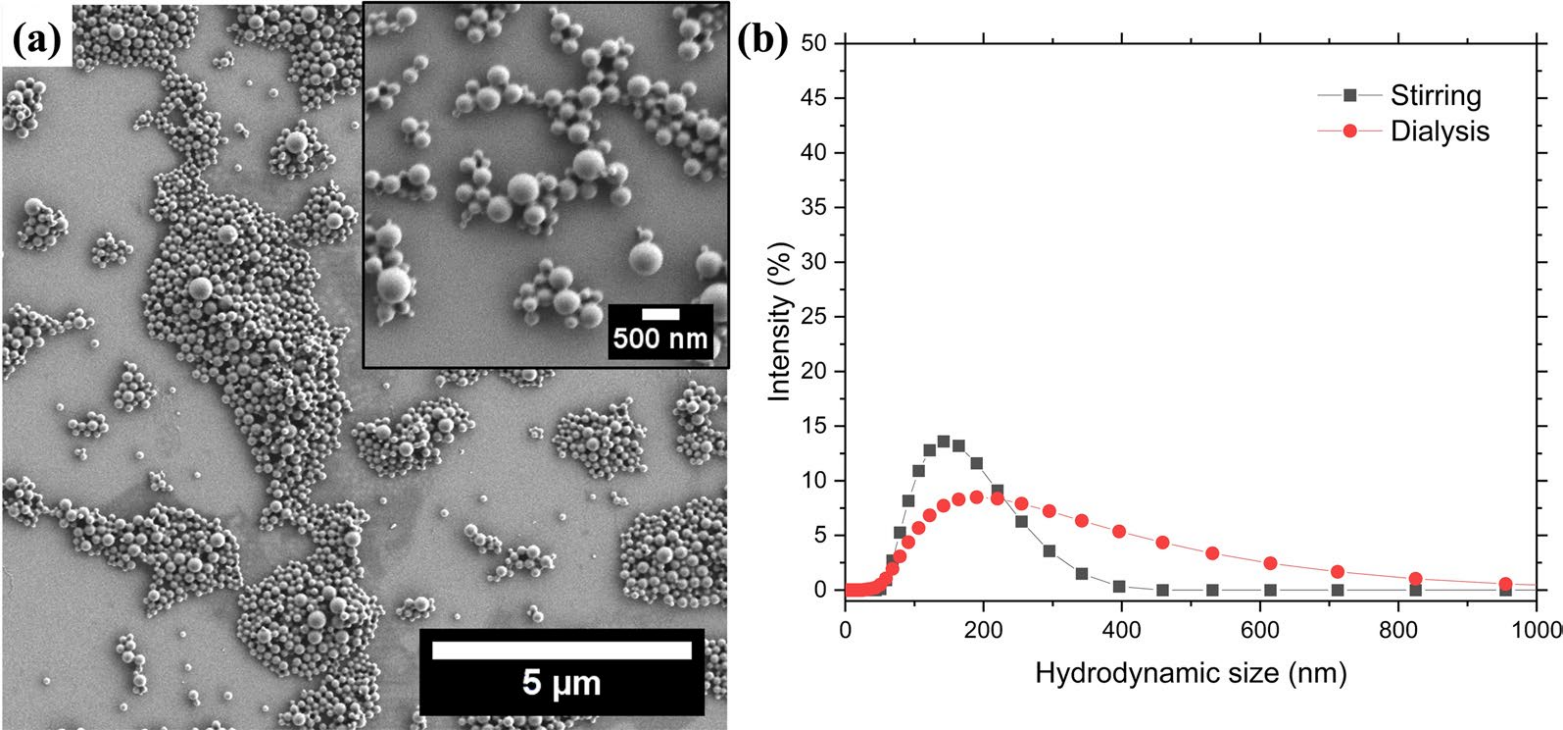
Spherical PLGA nanoparticles with on average a uniform size distribution were obtained by a double-emulsion solvent evaporation method. **a** SEM image of PLGA nanoparticles synthesized using standard conditions and evaporated through stirring. Inset corresponds to higher magnification micrograph of PLGA nanoparticles synthesized as described previously. **b** Intensity size distribution of PLGA nanoparticles synthesized with stirring or dialysis for solvent evaporation

A double-emulsion solvent evaporation method was used to synthesize PLGA nanoparticles using vitamin E-TPGS as emulsifier and stabilizer [49, 50]. Spherical PLGA nanoparticles with different size distributions were obtained after varying synthesis parameters (Fig. 2a and Fig. S1). The size distribution was influenced by the method used for solvent removal (Fig. 2b) and the parameters used during synthesis (Fig. S2). PLGA nanoparticles had a smaller mean hydrodynamic size when using stirring (Z_ave_ = 151.8 nm) for solvent removal relative to those obtained with dialysis (Z_ave_ = 255.4 nm). Solvent removal by dialysis also resulted in a broader size distribution relative to that obtained with stirring (Fig. 2b). The concentration of PLGA and vitamin E-TPGS, number of sonication cycles, and payload solvent significantly influenced the mean hydrodynamic size and polydispersity of PLGA nanoparticles (Fig. S2). A lower concentration of vitamin E-TPGS (0.1 wt %) yielded the largest mean hydrodynamic size, Z_ave_ > 1,000 nm (Fig. S2b). As shown in Fig. S2 h, a single sonication cycle increased both the hydrodynamic size (Z_ave_ = 627.8 nm) and polydispersity (PDI = 0.428) of PLGA nanoparticles relative to those synthesized using standard conditions (Z_ave_ = 155.3 nm and PDI = 0.172). The intensity size distribution was used to assess the stability of the PLGA nanoparticles in DI H_2_O, PBS, and DMEM/F12 complete media over time (Fig. S3). The mean hydrodynamic size was similar for PLGA nanoparticles dispersed in DI H_2_O and PBS up to 7 days (Table S1). After 12 days, PLGA nanoparticles in PBS had a larger mean hydrodynamic size (Z_ave_ = 250.4 nm) compared to those in DI H_2_O (Z_ave_ = 158.5 nm). The size distribution of PLGA nanoparticles in DMEM/F12 complete media was characterized by two peaks (Fig. S3C): a small one below 100 nm (< 3.0%) and a large one around 200 nm (> 15–20%). The presence of the smaller peak decreased the mean hydrodynamic size of PLGA nanoparticles to ~ 130 nm (Table S1). The zeta potential of PLGA nanoparticles remained unchanged after exposure to DMEM/F12 complete media for 24 h and 72 h. The average zeta potential of PLGA nanoparticles diluted in DI H_2_O was − 24.4 ± 1.9 mV compared to those diluted in DMEM/F12 complete media − 29.3 ± 4.3 mV and − 22.1 ± 0.1 mV, respectively.

**Fig. 3:**
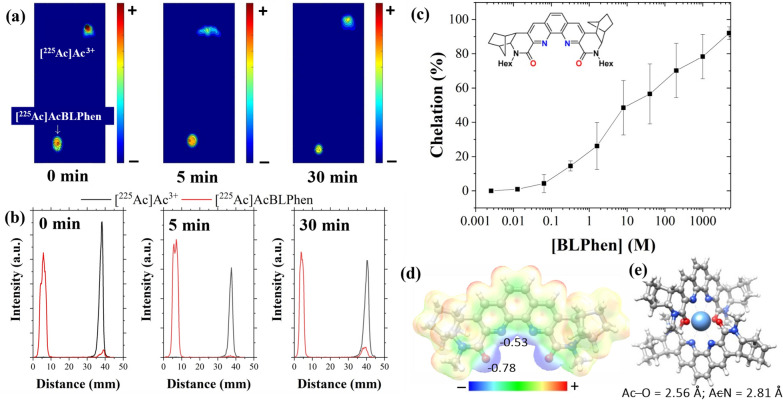
Understanding the chelation and coordination of [^225^Ac]Ac^3+^ by the lipophilic BLPhen ligand. **a** Autoradiographic images of [^225^Ac]Ac^3+^ and [^225^Ac]AcBLPhen at different times after co-mixing. Color scale shows different activities/intensities of [^225^Ac]Ac^3**+**^ and decay daughters with blue and red as the lowest and highest, respectively. **b** Histograms of autoradiographic images of [^225^Ac]Ac^3+^ and [^225^Ac]AcBLPhen at different times after co-mixing. **c** Chelation of [^225^Ac]Ac^3**+**^ as a function of BLPhen ligand concentration, 5 min after co-mixing. Insert corresponds to the chemical structure of the lipophilic BLPhen ligand.^47^. **d** Chemical structure of BLPhen with plotted electrostatic potential map (0.01 *e*/*a*_0_^3^ isovalue) and charges on O and N atoms obtained from natural population analysis. **e** Structure of the [^225^Ac][Ac(BLPhen)_2_]^3+^ complex optimized at the B3LYP-D3/LC/6–31 + G(d) level of theory. Color scheme: Ac, sky blue; C. grey; H, white; N, blue; O, red

Chelation of [^225^Ac]Ac^3+^ with a lipophilic BLPhen ligand was demonstrated using thin-layer chromatography and autoradiography. Free [^225^Ac]Ac^3+^ moves with the solvent front to the top of the plate, whereas [^225^Ac]AcBLPhen remains at the baseline (Fig. 3a and b). Lipophilic BLPhen ligand chelates [^225^Ac]Ac^3+^ rapidly after co-mixing both solutions at room temperature. The chelation efficiency is 92.0 ± 3.7% at 5 mM BLPhen ligand (n = 3); however, it decreases below 70% for concentrations < 200 µM (Fig. 3c). To gain structural insights into the coordination chemistry of [^225^Ac]Ac^3+^ with the BLPhen ligand, quantum chemical calculations were performed using DFT for the truncated version of BLPhen, where the long hexyl substituents were replaced by methyl groups. Based on a recent x-ray absorption fine structure spectroscopy (XAFS) study [72], pointing to the presence of a 1:2 local coordination in the Ln–BLPhen species formed in the organic phase, the calculations were performed for the cationic [^225^Ac][Ac(BLPhen)_2_]^3+^ complex. The electrostatic potential map of the BLPhen molecule clearly shows the highly electronegative regions formed by the amide oxygen (O_amide_) and phenanthroline nitrogen (N_phen_) groups constituting the ideal chelating sites suitable for [^225^Ac]Ac^3+^ binding and retention (Fig. 3d). These favorable binding interactions were confirmed by the calculated negative Gibbs free energy value for the displacement of nine water molecules from the Ac^3+^ aqua ion by two BLPhen ligands, according to reaction 1:

1 $$Ac{(H}_{2}O{)}_{9}^{3+}+2BLPhen\rightleftharpoons Ac(BLPhen{)}_{2}^{3+}+9{H}_{2}O, \Delta {G}_{aq}= -28.12\frac{kcal}{mol}$$

The DFT-optimized geometry of the resulting [^225^Ac][Ac(BLPhen)_2_]^3+^ complex shows that the Ac–O_amide_ bond distances are 0.25 Å shorter than the Ac–N_phen_ distances. This indicates that the amide oxygen atoms possess relatively stronger electron donating ability toward [^225^Ac]Ac^3+^ in comparison with the phenanthroline nitrogen atoms (Fig. 3e). To further probe these interactions, we performed NBO analysis of chemical bonding in the actinide complex. As expected, the calculated Wiberg bond indices, which serve as a measure of bond order, were higher for Ac–O_amide_ bonds (0.152) than for Ac–N_phen_ bonds (0.111). Additionally, the NBO analysis indicated that the formal polarized Ac–O_amide_ and Ac–N_phen_ dative bonds originated from a σ-type donation of electron density from the donor O and N lone pairs to the vacant acceptor orbitals of primarily 6d character on [^225^Ac]Ac^3+^.

**Fig. 4:**
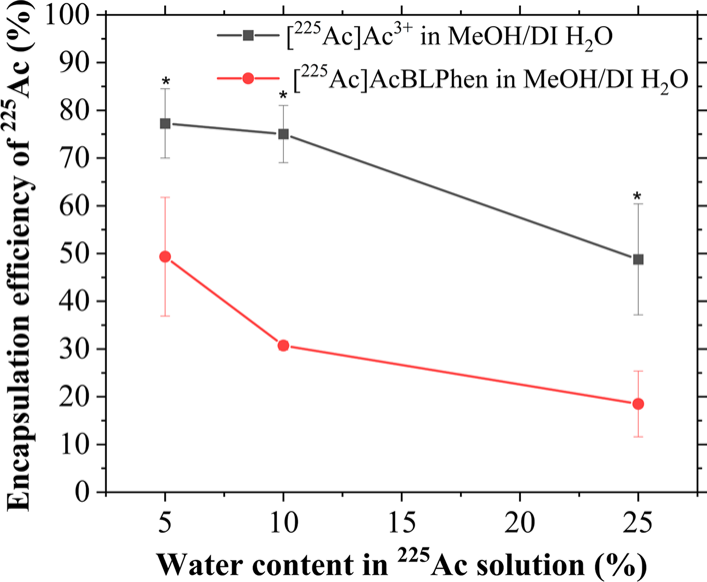
Encapsulation of [^225^Ac]Ac^3+^ within PLGA nanoparticles favors a hydrophobic environment. The encapsulation efficiency of [^225^Ac]Ac^3+^ within PLGA nanoparticles increased by decreasing the water content of the [^225^Ac]Ac^3+^ solution. The plot represents the encapsulation efficiency of [^225^Ac]Ac^3+^ within PLGA nanoparticles as free cations and when chelated to a BLPhen ligand, using a MeOH/DI H_2_O mixture with different water contents. Values and error bars correspond to the mean and standard error of three replicates, respectively. *P < 0.05 one-way ANOVA followed by Tukey multiple comparisons post-test

The encapsulation efficiency of La within PLGA nanoparticles increased with decreasing payload volume (1.27%, 0.67%, and 0.45% for 5 µL, 10 µL, and 20 µL, respectively). Co-mixing LaCl_3_ with a MOPS buffer (0.1 M, pH 7.2, 0.15 M NaCl) increased the La encapsulation efficiency to 53.2%. The encapsulation efficiency of [^225^Ac]Ac^3+^ followed a similar trend to that of La: It was less than 5% for [^225^Ac]Ac^3+^ dispersed in DI H_2_O; and it increased to 35.2 ± 8.1% after reconstituting [^225^Ac]Ac^3+^ in NH_4_OAc solution (0.1 M, pH 6.0). Encapsulation of [^225^Ac]Ac-macropa complex was less than 2% within PLGA nanoparticles. Chelation of [^225^Ac]Ac^3+^ to BLPhen ligand increased the encapsulation efficiency (> 20%) relative to free [^225^Ac]Ac^3+^ in DI H_2_O and [^225^Ac]Ac-macropa complex. The encapsulation of [^225^Ac]Ac^3+^ in a MeOH/DI H_2_O mixture was evaluated as a control for the encapsulation of [^225^Ac]AcBLPhen complex. The encapsulation efficiency of [^225^Ac]Ac^3+^ in a MeOH/DI H_2_O mixture was higher than that of [^225^Ac]AcBLPhen for the same water content (Fig. 4). Increasing the water content in the MeOH/DI H_2_O mixture decreased the encapsulation efficiency within PLGA nanoparticles for both [^225^Ac]Ac^3+^ and [^225^Ac]AcBLPhen (Fig. 4).

**Fig. 5:**
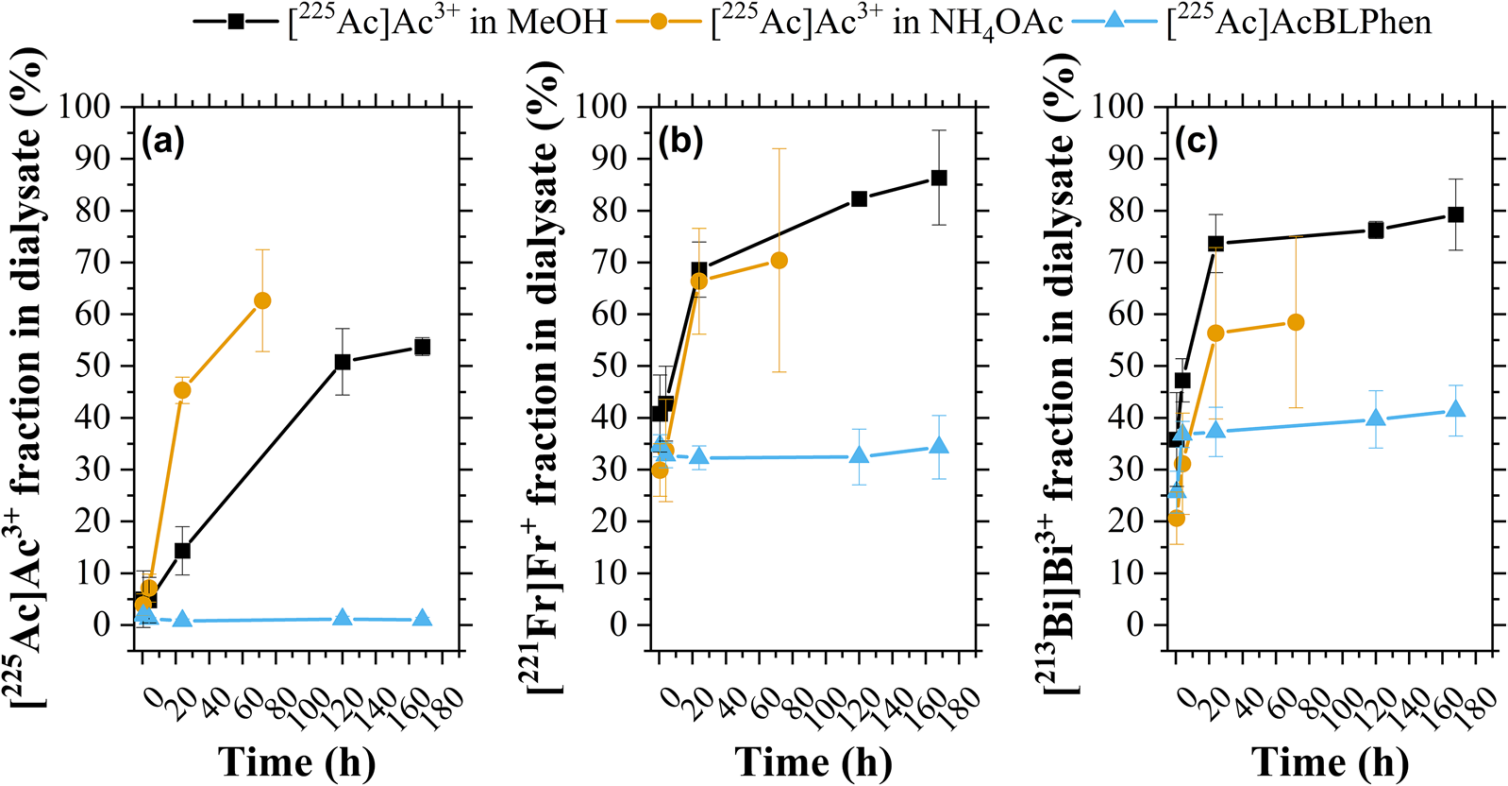
Enhanced retention of [^225^Ac]Ac^3+^ and its decay daughters, [^221^Fr]Fr^+^ and [^213^Bi]Bi^+^, was achieved by encapsulating [^225^Ac]AcBLPhen within PLGA nanoparticles. The fraction of **a** [^225^Ac]Ac^3+^, **b** [^221^Fr]Fr^+^, and **c** [^213^Bi]Bi^3+^ detected in the dialysate for PLGA nanoparticles encapsulating [^225^Ac]Ac^3+^ in a MeOH/DI mixture, [^225^Ac]Ac^3+^ in NH_4_OAc solution, and [^225^Ac]AcBLPhen. Values and error bars correspond to the mean and standard error of three replicates, respectively. The release of [^221^Fr]Fr^+^ and [^213^Bi]Bi^3+^ considers the fraction of activity originating from [^225^Ac]Ac^3+^ in the dialysate as defined by the equations in the supporting information

The retention of [^225^Ac]Ac^3+^ and its decay daughters, [^221^Fr]Fr^+^ and [^213^Bi]Bi^3+^, within PLGA nanoparticles was assessed by measuring the fraction of radioactivity in the dialysate. Water/oil/water emulsions with [^225^Ac]Ac^3+^ in a MeOH/DI mixture, [^225^Ac]AcBLPhen, and [^225^Ac]Ac^3+^ in NH_4_OAc as payload were transferred into dialysis cassettes and dialyzed against PBS. The water content in the MeOH/DI mixture used for [^225^Ac]Ac^3+^ and [^225^Ac]AcBLPhen was 5% (Fig. 4). Retention of [^225^Ac]Ac^3+^ within PLGA nanoparticles was influenced by the payload solution used during synthesis (Fig. 5a). PLGA nanoparticles encapsulating [^225^Ac]Ac^3+^ in NH_4_OAc displayed a substantial release of [^225^Ac]Ac^3+^ (45.3 ± 2.5%) after 24 h in dialysis (Fig. 5a). A controlled release of [^225^Ac]Ac^3+^ was observed when using the [^225^Ac]Ac^3+^ in a MeOH/DI H_2_O mixture as payload (Fig. 5a). This mixture resulted in a maximum of 53.7 ± 1.7% of [^225^Ac]Ac^3+^ found in the dialysate after 168 h (Fig. 5a). The release of [^225^Ac]Ac^3+^ was less than 2% when encapsulating [^225^Ac]AcBLPhen within PLGA nanoparticles (Fig. 5a). Release of ^221^Fr was similar for PLGA nanoparticles encapsulating [^225^Ac]Ac^3+^ in NH_4_OAc and [^225^Ac]Ac^3+^ in a MeOH/DI H_2_O mixture, > 60% after 24 h in dialysis (Fig. 5b). The fraction of [^221^Fr]Fr^+^ released from PLGA nanoparticles encapsulating [^225^Ac]AcBLPhen varied between 32 and 35% over time (Fig. 5b). Similar results were obtained for [^213^Bi]Bi^3+^: PLGA nanoparticles with [^225^Ac]Ac^3+^ in NH_4_OAc and [^225^Ac]Ac^3+^ in a MeOH/DI H_2_O mixture exhibited a greater release of [^213^Bi]Bi^3+^ than did PLGA nanoparticles encapsulating [^225^Ac]AcBLPhen (Fig. 5c).

**Fig. 6:**
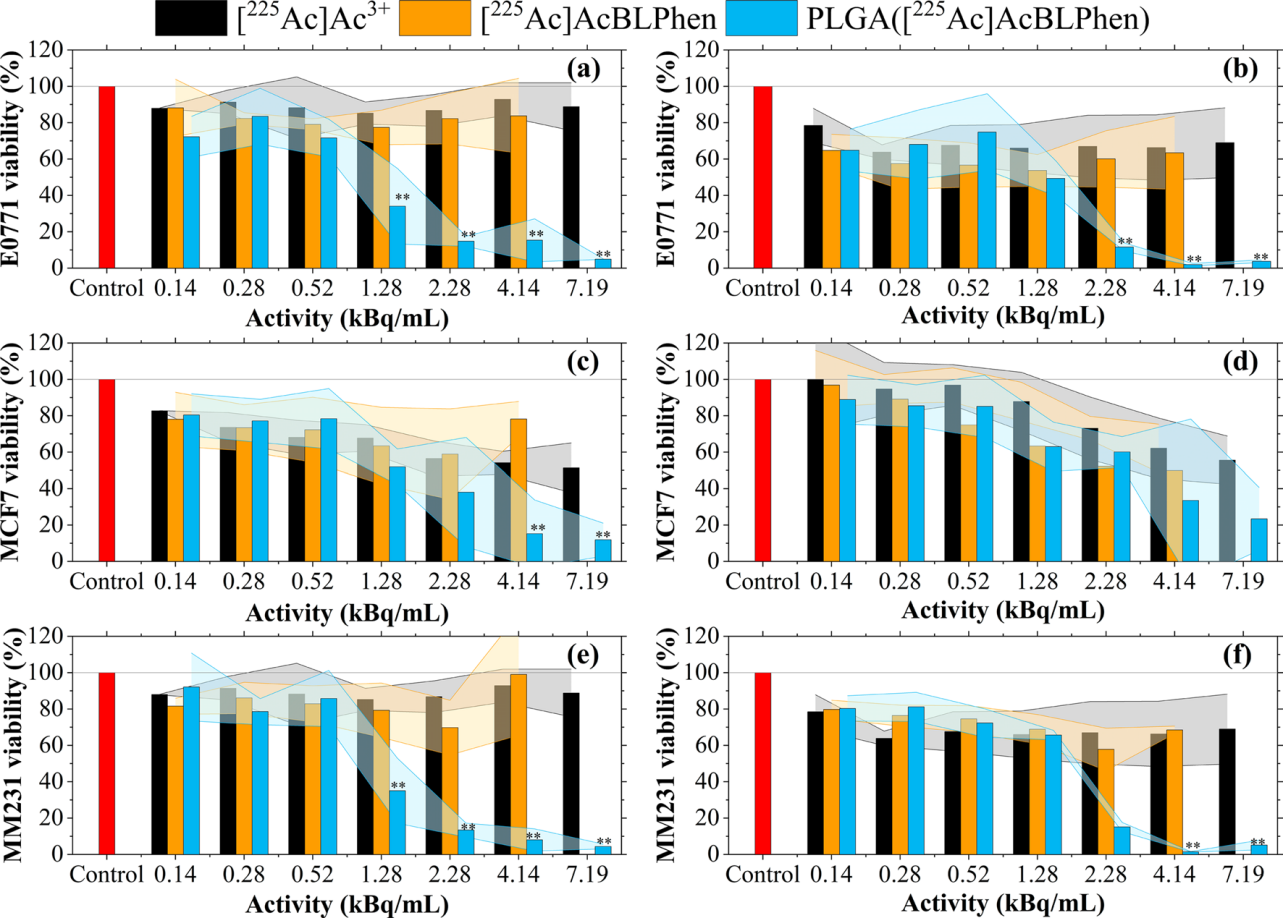
PLGA nanoparticles encapsulating [^225^Ac]AcBLPhen are cytotoxic to breast cancer cells. The viability of E0771, MCF-7, and MDA-MB-231 breast cancer cells after exposure for 24 h to free [^225^Ac]Ac^3+^, [^225^Ac]AcBLPhen, and PLGA nanoparticles encapsulating [^225^Ac]AcBLPhen. Cell viability relative to untreated cells was assessed (**a**, **c**, **e**) 1 h and (**b**, **d**, **f**) 72 h after exposure to [^225^Ac]Ac^3+^ using alamarBlue. Data is presented as the mean and standard deviation for at least three biological replicates. **P < 0.01 (relative to both free [^225^Ac]Ac^3+^ and [^225^Ac]AcBLPhen) one-way ANOVA followed by Tukey multiple comparisons post-test

The cytotoxic effect of PLGA nanoparticles encapsulating [^225^Ac]AcBLPhen was evaluated against free [^225^Ac]Ac^3+^ and [^225^Ac]AcBLPhen, hereafter referred to as controls. E0771, MCF-7, and MDA-MB-231 breast cancer cells were exposed to all [^225^Ac]Ac^3+^ solutions for 24 h. Cell viability was assessed 1 h and 72 h after exposure to different ^225^Ac activities. At 1 h post-exposure, the viability of E0771 (Fig. 6a) and MDA-MB-231 (Fig. 6e) cells decreased significantly when using activities greater than 1.3 kBq/mL delivered within PLGA nanoparticles. For MCF-7 cells, no significant difference in cell viability was observed between controls and PLGA nanoparticles when using activities below 4.1 kBq/mL (Fig. 6c). At 72 h post-exposure, the viability of E0771 cells was not significantly different between controls and PLGA nanoparticles for activities below 2.3 kBq/mL (Fig. 6b). For MDA-MB-231 cells, exposure to activities greater than 2.3 kBq/mL was required to observe a significant difference in viability between PLGA nanoparticles encapsulating [^225^Ac]AcBLPhen to controls (Fig. 6f). MCF-7 cell viability was not significantly different between PLGA nanoparticles and controls for the [^225^Ac]Ac^3+^ activities used in this work at 72 h post-exposure (Fig. 6d). Overall, it appears that the cell killing effect of [^225^Ac]Ac^3+^ is more pronounced when delivered to cells using the PLGA nanoparticles. The MDA-MB-231 and the E0771 cells showing significant cell death at 1.3 kBq/mL and 2.3 kBq/mL at 1 h. Persistent loss of viability was observed for these cell lines at doses above 2.3 kBq/mL at 72 h, while some recovery was noted at 1.3 kBq/mL. Interestingly, the MCF-7 cells showed significant cell death to PLGA nanoparticles encapsulating [^225^Ac]AcBLPhen at 4.1 kBq/mL and 7.2 kBq/mL when exposed for 1 h and these cells appear to show some recovery at 72 h.

**Fig. 7:**
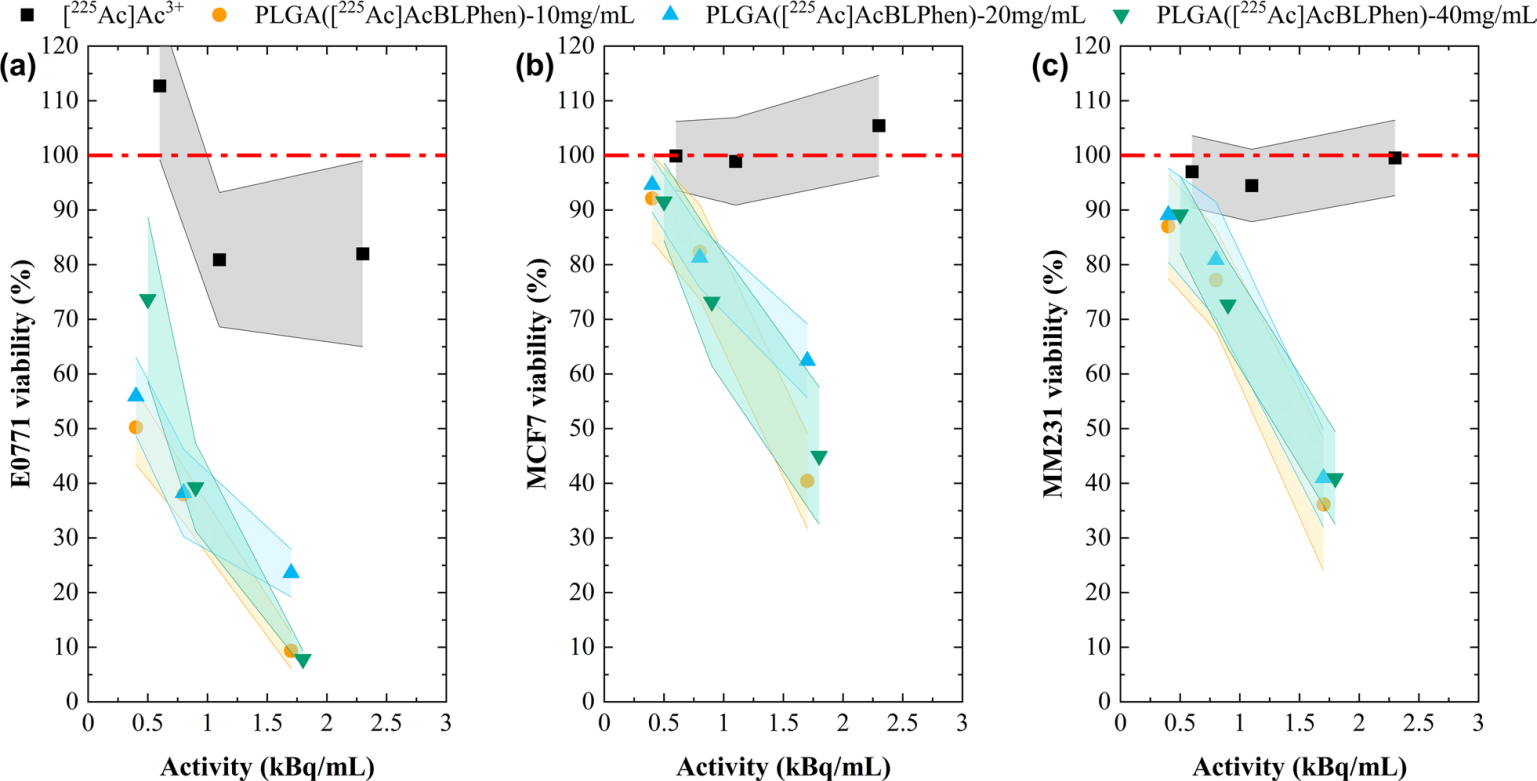
A similar cytotoxic effect was observed when [^225^Ac]Ac^3+^ was delivered with different concentrations of PLGA. Delivering [^225^Ac]Ac^3+^ with different concentrations of PLGA nanoparticles did not influence its cytotoxic effect on **a** E0771, **b** MCF-7, and **c** MDA-MB-231 cells. Cell viability after exposure to [^225^Ac]Ac^3+^ in PBS and PLGA nanoparticles encapsulating [^225^Ac]AcBLPhen at 10 mg/mL, 20 mg/mL, and 40 mg/mL for 24 h. Cell viability was assessed 1 h post-exposure to [^225^Ac]Ac^3+^ using alamarBlue assay relative to untreated cells. Cells were exposed to free [^225^Ac]Ac^3+^ (0.6 kBq/mL, 1.1 kBq/mL, and 2.3 kBq/mL), PLGA([^225^Ac]AcBLPhen) [10 mg/mL] (0.4 kBq/mL, 0.8 kBq/mL, and 1.7 kBq/mL), PLGA([^225^Ac]AcBLPhen) [20 mg/mL] (0.4 kBq/mL, 0.8 kBq/mL, and 1.7 kBq/mL), and PLGA([^225^Ac]AcBLPhen) [40 mg/mL] (0.5 kBq/mL, 0.9 kBq/mL, and 1.8 kBq/mL). Reported values correspond to the mean of 12 technical replicates and n = 1 experiment. Error bars show the relative error

Delivering similar [^225^Ac]Ac^3+^ activities with different concentrations of PLGA nanoparticles encapsulating [^225^Ac]AcBLPhen (e.g., 10 mg/mL, 20 mg/mL, and 40 mg/mL) did not significantly affect cell viability (Fig. 7). The viability of all cell lines exposed to PLGA nanoparticles encapsulating [^225^Ac]AcBLPhen was different from that of free [^225^Ac]Ac^3+^ when using activities greater than 0.8 kBq/mL (Fig. 7). E0771 were more sensitive to free [^225^Ac]Ac^3+^ and PLGA nanoparticles encapsulating [^225^Ac]AcBLPhen relative to MCF-7 and MDA-MB-231 cells. E0771 cell viability decreased below 80% and 40% after exposure to activities greater than 0.4 kBq/mL delivered as free [^225^Ac]Ac^3+^ and [^225^Ac]AcBLPhen encapsulated within PLGA nanoparticles, respectively (Fig. 7a). As shown in Fig. S4, E0771 cells had lower internalization and uptake of fluorescent PLGA-Cy5 nanoparticles relative to that of MDA-MB-231 cells (Fig. S5). The damage induced by α-particles was evaluated by measuring γ-H2AX foci, a marker of DNA double-strand breaks (Fig. S6). Cells were exposed to free [^225^Ac]Ac^3+^ and PLGA nanoparticles encapsulating [^225^Ac]AcBLPhen for 24 h. When using free [^225^Ac]Ac^3+^, E0771 cells exhibited more γ-H2AX foci indicating that they are more susceptible to DNA damage than MCF-7 and MDA-MB-231 cells (Figs. S7a, S8a, and S9a). For E0771 cells, the number of foci per nucleus increased with greater activities of free [^225^Ac]Ac^3+^ (Figs. S6 and S7a). For PLGA nanoparticles, this trend was not as significant compared to that of free [^225^Ac]Ac^3+^ because the number of viable cells decreased drastically at the highest [^225^Ac]Ac^3+^ activities (Fig. 6). The effect of exposure time on E0771 and MCF-7 cell viability was evaluated with free [^225^Ac]Ac^3+^ and PLGA nanoparticles encapsulating [^225^Ac]AcBLPhen. Viability analysis 24 h post-exposure showed similar viability of E0771 cells for the different activities and incubation times when using free [^225^Ac]Ac^3+^ (Fig. S10a). E0771 cells exposed to free [^225^Ac]Ac^3+^ (> 2 kBq/mL) for 24 h showed a slight decrease in viability when evaluated after 48 h (Fig. S10b). No appreciable effects were noted for shorter exposures to free [^225^Ac]Ac^3+^ under these conditions (Fig. S10b). By comparison the incubation of E0771 cells with PLGA nanoparticles encapsulating [^225^Ac]AcBLPhen for 24 h shows a clear impact to cell viability: a reduction of viability to 43.7% and 34.7% when exposed to 1.6 kBq/mL at 24 h and 48 h post-exposure, respectively (Fig. S10C and S10D). Exposure to 3.1 kBq/mL for 24 h decreased E0771 cell viability to 8.9% and 4.3% at each time post-exposure (Figs. S10c and S10d).

For MCF-7, little change in viability was observed when assessing it 24 h post-exposure to free [^225^Ac]Ac^3+^ for all doses and times (Fig. S11a). At 48 h, free [^225^Ac]Ac^3+^ resulted in cytotoxic effects when using 4.0 kBq/mL with viability dropping to 67.2% (Fig. S11b). In contrast, the viability of MCF-7 cells decreased significantly after incubation with PLGA nanoparticles at 3.1 kBq/mL (Fig. S11c and S11d). At 24 h and 48 h, a decrease in viability greater than 75% was observed in cells exposed to highest activity (3.1 kBq/mL) for 24 h. A lower activity of 1.6 kBq/mL showed a further decrease in MCF-7 cell viability when tested at 48 h (54.4%) from that observed 24 h post-treatment (78.8%) (Fig. S11c and S11d).

## Discussion

A double-emulsion solvent evaporation method was adapted for the synthesis of PLGA nanoparticles in a radiological laboratory. The main adaptations include using small reagent volumes and a cup-horn sonicator to generate emulsions. Spherical PLGA nanoparticles are obtained with this method and their mean size and size distribution can be tailored by adjusting various parameters (Fig. S2). Of these parameters, the solvent removal method and the emulsifier concentration during sonication had a significant impact on the distribution and mean size of PLGA nanoparticles, respectively (Fig. 2b and S2b). As a solvent removal method, dialysis was also used for removal of free radionuclides and facilitate buffer exchange into PBS. It is expected that the sample will achieve a purification factor equal to the volume ratio between sample and dialysate (approximately 1:180–200). Additionally, the diffusion rate through the membrane is affected by dialysis parameters including temperature and stirring in addition to molecule/contaminant characteristics such as concentration and molecular weight. Because dialysis was conducted at room temperature with constant stirring (120 rpm), it is anticipated that free radionuclides—having a small molecular weight and a relatively low concentration—will diffuse readily through the dialysis membrane. Additionally, the type of dialysis device used—Slide-A-Lyzer cassettes—will also improve the diffusion rate of radionuclides and organic solvents. The enhancement of diffusion rate is related with the flat configuration of the dialysis cassettes, which facilitates that the molecules are close to the membrane. Based on these factors, 4-h dialysis with constant stirring at room temperature is sufficient time for the diffusion of radionuclides and organic solvents from the sample to the dialysate. Low concentration of emulsifier is not desirable due to a large mean hydrodynamic size (Z_ave_ > 1000 nm), broad size distribution, and incomplete particle formation (Fig. S1d and S1f). The increase in size and partial formation of PLGA nanoparticles was due to a lower fraction of emulsifier molecules than that required for controlled particle growth. Increasing the concentration of emulsifier contributes to a smaller size and more uniform size distribution (Fig. S2b). These characteristics are required for translation of these PLGA nanoparticles into future in vivo studies [36, 37]. Adaptation of a double-emulsion solvent evaporation method for radiological synthesis of PLGA nanoparticles resulted in particles with a similar size distribution to those obtained previously [49, 50]. PLGA nanoparticles were found to be stable in DI H_2_O, PBS, and DMEM/F12 complete media over multiple ^225^Ac half-lives (Fig. S3). Only incubation in PBS resulted in appreciable broadening of the size distribution after 14 days (Fig. S3b). A slight broadening of the main hydrodynamic size peak was observed for the nanoparticles incubated with complete media containing 10% serum relative to those in DI H_2_O (Fig. S3c). No change in the peak shape was observed for PLGA nanoparticles diluted in DI H_2_O or complete cell culture media over the time course of the experiment. The presence of vitamin E TPGS may contribute to the stability of PLGA nanoparticles in the different solvents as well as prevents the surface adsorption of proteins from DMEM/F12 complete media.

Experiments using La^3+^ as a surrogate for [^225^Ac]Ac^3+^ showed that encapsulation of free La^3+^ is not suitable for evaluation in cell culture studies (< 2%). Reconstituting La cations with MOPS buffer significantly increased the encapsulation of La^3+^ within PLGA nanoparticles, as evidenced in our previous work [49]. Building from these results, the encapsulation of [^225^Ac]Ac^3+^ in PLGA nanoparticles was explored as (1) a free cation; (2) reconstituted in NH_4_OAc, and (3) chelated to a ligand. Encapsulation of free [^225^Ac]Ac^3+^ in DI H_2_O was < 5% because [^225^Ac]Ac^3+^ remained in the aqueous phase and was thus excluded from the hydrophobic environment of PLGA nanoparticles. Reconstituting [^225^Ac]Ac^3+^ with NH_4_OAc increased its encapsulation efficiency to 59.4 ± 3.1% owing to the formation of [^225^Ac]Ac-acetate complexes. Chelation of [^225^Ac]Ac^3+^ to the hydrophilic macropa chelator (LogP = 0.222 ± 0.7 calculated using Advanced Chemistry Development (ACD/Labs) Software V11.02) [73] did not increase the encapsulation efficiency compared to that of free [^225^Ac]Ac^3+^ in DI H_2_O. Like free [^225^Ac]Ac^3+^, [^225^Ac]Ac-macropa complexes had poor encapsulation efficiency because of the hydrophilic nature of the complex. Chelation of [^225^Ac]Ac^3+^ by a lipophilic BLPhen ligand (LogP = 10.3 ± 1.3 calculated using Advanced Chemistry Development (ACD/Labs) Software V11.02) [73] was therefore investigated as an alternative to improve the encapsulation efficiency within PLGA nanoparticles. Successful chelation of [^225^Ac]Ac^3+^ by lipophilic BLPhen ligand (> 90%) was achieved at room temperature for ligand concentrations greater than 5 mM (Fig. 3c). This is the first instance of a BLPhen ligand binding [^225^Ac]Ac^3+^ and opens the potential of this ligand for TAT. Lower BLPhen ligand concentrations caused a drop in chelation efficiency. This decrease in efficiency may be associated with the rigidity of the BLPhen ligand, which has been shown to impact the affinity of the BLPhen ligand for lanthanide cations [46, 47]. Ligand-lanthanide complexes with both 1:1 and 2:1 stoichiometries have been observed in the organic phase for lipophilic BLPhen ligand [46]. Computational analyses also showed that BLPhen coordinates to [^225^Ac]Ac^3+^ in a tetradentate fashion to form a 2:1 complex by exploiting a rigid and preorganized binding pocket. It is anticipated that modifying the BLPhen ligand structure will alter its rigidity which may increase its chelation efficiency for [^225^Ac]Ac^3+^ at lower ligand concentrations.

The lipophilic nature of the [^225^Ac]AcBLPhen complex was expected to: (i) decrease its water partitioning during nanoparticle synthesis, (ii) improve the encapsulation of [^225^Ac]Ac^3+^ within PLGA nanoparticles, and (iii) minimize [^225^Ac]Ac^3+^ release from PLGA nanoparticles. An encapsulation efficiency of 49.3 ± 12.4% was obtained with [^225^Ac]AcBLPhen when the water content in the mixture was 5% (Fig. 4). The encapsulation efficiency of [^225^Ac]Ac^3+^ in a DI H_2_O/MeOH mixture was studied as a control for the lipophilic BLPhen ligand. Actinium-225 in this mixture had a higher encapsulation efficiency within PLGA nanoparticles relative to that of the lipophilic BLPhen ligand (Fig. 4). Increasing the water content in the [^225^Ac]Ac^3+^ DI H_2_O/MeOH mixture to 25% resulted in a decrease in encapsulation efficiency to 48.8 ± 11.6% (Fig. 4). The lower [^225^Ac]Ac^3+^ encapsulation efficiency paralleled an increase in water content in the payload mixture, which is associated with a decrease in miscibility with the organic phase [74]. It is hypothesized that transmetalation of ^225^Ac during emulsification causes the lower encapsulation efficiency for [^225^Ac]AcBLPhen with respect to [^225^Ac]Ac^3+^ in DI H_2_O/MeOH. The transmetalation of ^225^Ac during emulsification could be caused by a lack of stability of the [^225^Ac]AcBLPhen and unfavorable reaction conditions. The reaction conditions could also promote the precipitation of the BLPhen complexes and, thus, the transmetalation of ^225^Ac. The encapsulation efficiency of [^225^Ac]Ac^3+^ within PLGA nanoparticles was higher than that reported for liposomes encapsulating [^225^Ac]Ac-DOTA (< 10%) [22, 23]. Polymersomes passively loaded with ^225^Ac using ionophore- or tropolone-mediated mechanisms reported a higher encapsulation efficiency (> 60%) than those obtained in this work [26, 28]. Additionally, similar encapsulation efficiency was obtained by loading [^177^Lu]Lu-DOTA-TATE into preformed PLGA nanoparticles (> 60%) [43, 44]. Overall, active encapsulation of [^225^Ac]Ac^3+^ within PLGA nanoparticles yielded similar encapsulation efficiencies to those obtained with passive encapsulation into preformed polymeric nanoparticles.

Retention of [^225^Ac]Ac^3+^ within PLGA nanoparticles was enhanced by its chelation to a BLPhen lipophilic ligand and by the hydrophobic composition of the payload solution (Fig. 5A). The [^225^Ac]Ac^3+^ reconstituted with NH_4_OAc experienced a burst release from PLGA nanoparticles due to the combined effects of PLGA nanoparticle hydrolysis, surface adsorption of [^225^Ac]Ac^3+^, and the rapid diffusion of encapsulated [^225^Ac]Ac^3+^ through the polymeric matrix into the dialysate [75]. A controlled release of [^225^Ac]Ac^3+^ from PLGA nanoparticles was obtained after encapsulation of free [^225^Ac]Ac^3+^ in a MeOH/DI H_2_O mixture. The extent of hydrophobicity within the PLGA nanoparticles—determined by the amount of water present in the [^225^Ac]Ac^3+^ solvent—gives rise to differences in release rates. A lower water content may alter the rate of diffusion through water pores and thus the rate of PLGA hydrolysis [75]. PLGA nanoparticles encapsulating [^225^Ac]AcBLPhen retained 98% of [^225^Ac]Ac^3+^ over time (Fig. 5a). The enhanced retention of [^225^Ac]Ac was due to the stability and lipophilic nature of the [^225^Ac]AcBLPhen complex and the low water content within these PLGA nanoparticles. The lack of a burst release from [^225^Ac]Ac^3+^ suggests that the [^225^Ac]AcBLPhen complex is not adsorbed onto the outer particle surface during synthesis [43]. It is expected that the release of [^225^Ac]Ac^3+^ may increase when challenged with biologically relevant media like serum or cerebrospinal fluid under dynamic in vivo conditions [45, 76]. The release of radionuclides in biologically relevant media may also depend on the labeling conditions and the transchelation of radionuclides by proteins and competing cations [45, 77].However, as observed in this study the cytotoxic effect of PLGA([^225^Ac]AcBLPhen) nanoparticles incubated with breast cancer cells in the presence of 10% serum was not adversely impacted when compared to [^225^Ac]Ac^3+^ alone. Overall, the retention of [^225^Ac]Ac^3+^ within PLGA nanoparticles encapsulating [^225^Ac]AcBLPhen was similar to that obtained with zwitterionic liposomes (88% over 30 days) and polymersomes (> 92% after 48 h) [22, 23, 26, 28].A high release of decay daughters, [^221^Fr]Fr^+^ and [^213^Bi]Bi^3+^, from PLGA nanoparticles was obtained after encapsulation of [^225^Ac]Ac^3+^ in a MeOH/DI H_2_O mixture and in an NH_4_OAc solution (Fig. 5b and 5c). These results were expected owing to the relationship between particle size and the retention of decay daughters within polymeric nanoparticles [22, 23, 26, 28]. The adsorption of [^225^Ac]Ac^3+^ onto the nanoparticle surface combined with an increased rate of PLGA hydrolysis would in turn increase the release of decay daughters into the dialysate [26]. The adsorption and implantation of decay daughters into adjacent PLGA nanoparticles was not significant under the experimental conditions tested (i.e., the nanoparticle concentration was approximately 8 mg/mL within the dialysis cassette) [78]. It is hypothesized that the implantation into adjacent nanoparticles is negligible because of the low electron density and amorphous structure of PLGA nanoparticles [79]. Encapsulating [^225^Ac]AcBLPhen within PLGA nanoparticles decreased the release of [^221^Fr]Fr^+^ and [^213^Bi]Bi^3+^ into the dialysate to 33% and 44%, respectively. The similar encapsulation efficiency of [^221^Fr]Fr^+^ and [^213^Bi]Bi^3+^ could be attributed to their chemical properties and origin. The transport of [^213^Bi]Bi^3+^ through the membrane is potentially slower than that of [^221^Fr]Fr^+^, whereas the majority of [^213^Bi]Bi^3+^ found in the dialysate may originate from [^221^Fr]Fr^+^.^33^ The enhanced retention of [^221^Fr]Fr^+^ and [^213^Bi]Bi^3+^ within PLGA nanoparticles may be explained by a random distribution of [^225^Ac]AcBLPhen within each nanoparticle [26], which was likely achieved as a result of the active loading during synthesis. Future research will evaluate the implantation and potential complexation of decay daughters by adjacent PLGA nanoparticles and BLPhen ligands following the experimental setup used by Kozempel et al. [78].

Exposure of MCF-7, MDA-MB-231, and E0771 cells to PLGA nanoparticles encapsulating [^225^Ac]AcBLPhen significantly decreased their viability compared with controls (Fig. 6). The high cytotoxic effect of PLGA nanoparticles encapsulating [^225^Ac]AcBLPhen is associated with their spatial distribution within the well and their uptake into cells. The accumulation of PLGA([^225^Ac]AcBLPhen) around cancer cells will increase the dose delivered and, thus, their cytotoxicity. The quantitative retention of [^225^Ac]Ac^3+^ and the partial retention of decay daughters within PLGA nanoparticles is enhanced by the cellular uptake of PLGA nanoparticles, increasing their cell killing potential [18]. Fluorescent PLGA-Cy5 nanoparticles accumulated around E0771 and MDA-MB-231 cells and were internalized into the cytoplasm after 2 h of incubation (Figs. S4 and S5). Noticeably, MDA-MB-231 cells appeared to internalize the PLGA nanoparticles to a greater extent than E0771 cells. Accumulation of nanoparticles around and within the cancer cells increases the resultant DNA damage. DNA damage was determined by measuring γ-H2AX foci a marker of double-strand breaks caused, in this study, by α-particles. Higher concentrations of [^225^Ac]Ac^3+^ increased the number of γ-H2AX foci per nucleus for both [^225^Ac]Ac^3+^ and PLGA nanoparticles encapsulating [^225^Ac]AcBLPhen. This trend was clearly observed for free [^225^Ac]Ac^3+^ and to a lesser extend with PLGA nanoparticles. The differences in viability and DNA damage between cells may be associated with their sensitivity to α-particles [67] and the rate of PLGA nanoparticle uptake and internalization [80]. Additionally, counting of γ-H2AX foci was challenging with PLGA nanoparticles encapsulating [^225^Ac]AcBLPhen because there was a lower fraction of viable cells available. These remaining cells likely had lower amounts of DNA damage which allowed them to remain adhered to the tissue culture surface.

The concentration of PLGA nanoparticles in solution did not induce cytotoxic effects in the human and murine breast cancer cells confirming that PLGA is a biocompatible delivery vehicle (Fig. 7) [39]. In this study, the relative fraction of [^225^Ac]AcBLPhen within PLGA nanoparticles determines the cytotoxic effect since the amount of fluorescent PLGA-Cy5 nanoparticles internalized at low and high concentrations was not significantly different for a given cell line (Fig. S4 and S5). It is anticipated that both free [^225^Ac]Ac^3+^ and [^225^Ac]AcBLPhen will remain in solution and uniformly distributed over the well volume, decreasing the dose delivered to the cells which are attached to the bottom of the plate. The exposure time to PLGA nanoparticles encapsulating [^225^Ac]AcBLPhen correlated with increasing cell death because the cumulative dose delivered to the cells increased over time. Exposure to the highest activity [^225^Ac]AcBLPhen within PLGA nanoparticles, for the longest incubation time, resulted in the greatest decrease in cell viability for both E0771 and MCF-7 (Figs. S10 and S11). PLGA nanoparticles encapsulating [^225^Ac]AcBLPhen have the potential to treat solid tumors and micrometastases by either intratumoral injection or active targeting after modifying PLGA nanoparticles with targeting vectors that recognize unique or overexpressed receptors on cancer cells.

## Conclusions

PLGA nanoparticles were evaluated as a biocompatible delivery platform for [^225^Ac]Ac^3+^ in TAT. A double-emulsion solvent evaporation method was adapted for radiological synthesis, decreasing the generation of radioactive waste, and mitigating radioactive contamination because of the aerosolization of radionuclides. The adapted synthesis method resulted in PLGA nanoparticles with a mean particle size of ~ 150 nm when standard conditions were used. The size and size distribution are easily tailored by adjusting various synthesis parameters. Encapsulation of free [^225^Ac]Ac^3+^ and [^225^Ac]Ac-macropa was low because of their hydrophilic nature. A high encapsulation efficiency of free [^225^Ac]Ac^3+^ in DI/MeOH mixture and in NH_4_OAc solution within PLGA nanoparticles was achieved by tailoring the hydrophobicity of the payload mixture and chelating [^225^Ac]Ac^3+^ with a lipophilic ligand. Encapsulating free [^225^Ac]Ac^3+^ within PLGA nanoparticles resulted in a burst release of [^225^Ac]Ac^3+^ and a low retention of its decay daughters. Chelation of [^225^Ac]Ac^3+^ to a lipophilic BLPhen ligand enhanced the retention of [^225^Ac]Ac^3+^ and its decay daughters within PLGA nanoparticles. The enhanced radionuclide retention combined with the targeted delivery of [^225^Ac]Ac^3+^ to cells—facilitated by nanoparticle deposition on the cell surface and subsequent internalization by breast cancer cells—resulted in an increased cytotoxic effect of PLGA nanoparticles encapsulating [^225^Ac]AcBLPhen relative to controls. These results demonstrate that PLGA nanoparticles are a promising delivery platform for [^225^Ac]Ac^3+^ in TAT. This study indicates that PLGA nanoparticles have the potential to be combined with other ligands that are not suitable for in vivo delivery of radionuclides owing to their low binding affinity, poor solubility in water, or low stability in biological environments. Therefore, PLGA nanoparticles offer a new opportunity to repurpose these ligands for TAT with the synergistic benefit of enhancing encapsulation and retention of radionuclides within a biocompatible delivery platform. Further optimization of size distribution, radionuclide encapsulation, and bioconjugation is required for successful translation of radioactive PLGA nanoparticles to pre-clinical experiments.

### Supplementary Information

Supplementary Material 1

## Data Availability

The datasets generated and/or analyzed during the current study are available from the corresponding author upon reasonable request.
