# Supplementary material for: Development of 225Ac-doped biocompatible nanoparticles for targeted alpha therapy
Source: J Nanobiotechnology. 2024 Jun 2;22:306. doi: 10.1186/s12951-024-02520-6 (PMC11145892; doi:10.1186/s12951-024-02520-6)
Supplement: Supplementary file 1 — Supplementary Material 1 [file 12951_2024_2520_MOESM1_ESM.docx]

**SUPPORTING INFORMATION**

**Equations:**

- Encapsulation efficiency (EE)

$$\boldsymbol{EE (\%)=}\left[ \frac{\boldsymbol{A}_{\boldsymbol{PLGA}}}{\boldsymbol{A}_{\boldsymbol{PLGA}}\boldsymbol{+}\boldsymbol{A}_{\boldsymbol{Supernatant}}\boldsymbol{+}\boldsymbol{A}_{\boldsymbol{Tube}}} \right]\boldsymbol{\times100}$$

A_PLGA_, A_Supernatant_, and A_Tube_ correspond to the ^225^Ac activity within the PLGA nanoparticle suspension, supernatant, and Eppendorf tube, respectively.

- Radionuclide retention

$$\boldsymbol{Ac}_{\boldsymbol{released}}\boldsymbol{(\%)=}\left[ \frac{\boldsymbol{A}_{\boldsymbol{dialysate}}}{\boldsymbol{A}_{\boldsymbol{0}}\boldsymbol{e}^{\boldsymbol{-}\boldsymbol{\lambda t}}} \right]\boldsymbol{\times}\boldsymbol{100}$$

A_dialysate_, A_0_, and t correspond to the [^225^Ac]Ac^3+^ activity in the dialysate, initial [^225^Ac]Ac^3+^ activity in the cassette, and time in dialysis, respectively.

$$\boldsymbol{Fr}_{\boldsymbol{released}}\boldsymbol{(\%)=}\left[ \frac{\boldsymbol{A}_{\boldsymbol{dialysate}}\boldsymbol{-} \boldsymbol{A}_{\boldsymbol{Ac}}}{\boldsymbol{A}_{\boldsymbol{0}}\boldsymbol{e}^{\boldsymbol{-}\boldsymbol{\lambda t}}} \right]\boldsymbol{\times}\boldsymbol{100}$$

$$\boldsymbol{A}_{\boldsymbol{dialysate}}\boldsymbol{(}\boldsymbol{nCi}\boldsymbol{)=}\left[ \frac{\boldsymbol{A}_{\boldsymbol{measured}}\boldsymbol{\times}\boldsymbol{t}\boldsymbol{\times}\boldsymbol{\lambda}}{\boldsymbol{1}\boldsymbol{-}\boldsymbol{e}^{\boldsymbol{-}\boldsymbol{\lambda t}}} \right]\boldsymbol{\times}\boldsymbol{100}$$

A_dialysate_, A_Ac,_ A_0_, and t correspond to the [^221^Fr]Fr^+^ activity in the dialysate, [^225^Ac]Ac^3+^ activity in the dialysate, initial [^225^Ac]Ac^3+^ activity in the cassette, and time in dialysis, respectively. Owing to the short half-life of ^221^Fr, the activity measured in the dialysate (A_measured_) must be corrected by decay.

$$\boldsymbol{Bi}_{\boldsymbol{released}}\boldsymbol{(\%)=}\left[ \frac{\boldsymbol{A}_{\boldsymbol{dialysate}}\boldsymbol{-} \boldsymbol{A}_{\boldsymbol{Ac}}}{\boldsymbol{A}_{\boldsymbol{0}}\boldsymbol{e}^{\boldsymbol{-}\boldsymbol{\lambda t}}} \right]\boldsymbol{\times}\boldsymbol{100}$$

A_dialysate_, A_Ac,_ A_0_, and t correspond to the [^213^Bi]Bi^3+^ activity in the dialysate, [^225^Ac]Ac^3+^ activity in the dialysate, initial [^225^Ac]Ac^3+^ activity in the cassette, and time in dialysis, respectively.

**
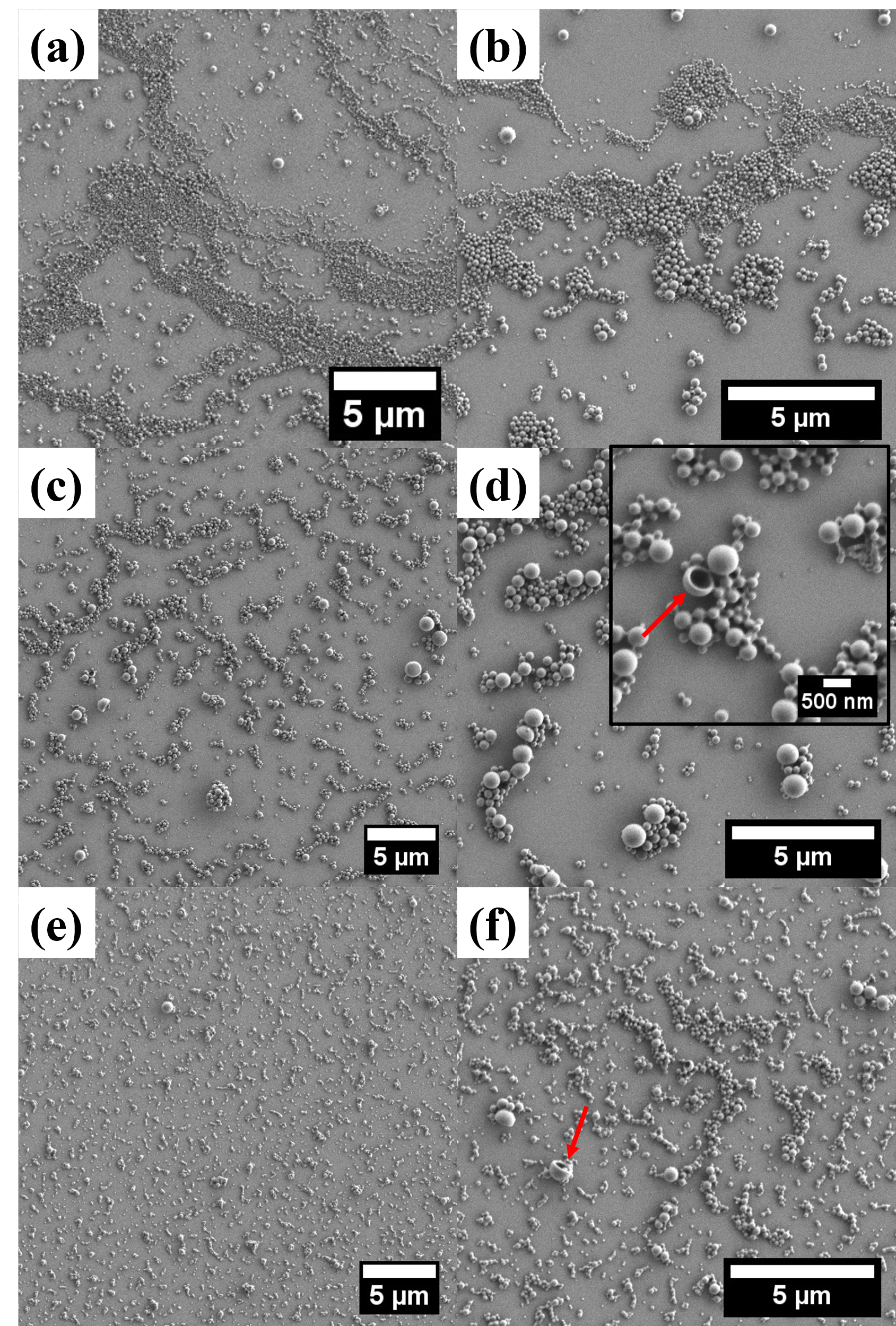
**

Figure S. 1. Spherical PLGA nanoparticles can be obtained with various size distributions by altering synthesis parameters. Representative SEM images of PLGA nanoparticles synthesized with standard conditions while the concentration of vitamin E-TPGS and the number of sonication cycles are varied. PLGA nanoparticles synthesized with (a, b) 1 wt % of vitamin E-TPGS and two sonication cycles, (c, d) 0.1 wt % of vitamin E-TPGS and two sonication cycles, and (e, f) 0.5 wt % of vitamin E-TPGS and one sonication cycle. Arrows (red) indicate larger PLGA nanoparticles were not completely formed or that have burst/collapsed during synthesis. Inset corresponds to a higher magnfication micrograph of PLGA nanoparticles synthesized with 0.1 wt % of vitamin E-TPGS and two sonication cycles.





Figure S. 2. The size distribution of PLGA nanoparticles can be tailored by adjusting synthesis parameters. Influence of (a) PLGA concentration, (b) TPGS concentration, (c) payload volume, (d) volume fraction between payload and EtOAc, (e) volume fraction between EtOAc and TPGS, (f) TPGS concentration in quench bath, (g) sonication time, (h) sonication cycles, and (i) payload solvent on the mean hydrodynamic size and polydispersity of PLGA nanoparticles. PLGA nanoparticles were synthesized using a double-emulsion solvent evaporation method. One parameter was varied at a time while the others were kept constant based on the standard conditions.





Figure S. 3. PLGA nanoparticles maintained a similar hydrodynamic size in different media. Stability of PLGA nanoparticles in (a) dionized water, (b) phosphate buffered saline, and (c) DMEM/F12 complete media. Intensity size distribution over time after maintaining the PLGA nanoparticles at 4ºC between measurements. Inset in (c) shows the presence of small intensity peak from DMEM/F12 complete media.

Table S. 1 PLGA nanoparticles suspended in complete culture media maintain similar size and size distribution over time. Mean hydrodynamic size (Z_ave_) and polydispersity (PDI) of PLGA nanoparticles in different media over time. PLGA nanoparticles remained stable in DI H_2_O, whereas a slight aggregation was observed after 14 days in PBS.

|  | **Z_ave_ (nm) [PDI]** | | | | |
| --- | --- | --- | --- | --- | --- |
| **Time (d)** | **0** | **1** | **3** | **7** | **14** |
| **DI H_2_O** | 167.7 [0.127] | 170.4 [0.113] | 170.2 [0.096] | 166.1 [0.107] | 158.5 [0.078] |
| **PBS** | 168.4 [0.129] | 167.6 [0.107] | 168.3 [0.110] | 166.2 [0.082] | 250.4 [0.253] |
| **DMEM/F12** | 125.9 [0.451] | 128.4 [0.373] | 130.8 [0.340] | 124.7 [0.434] | 125.5 [0.382] |


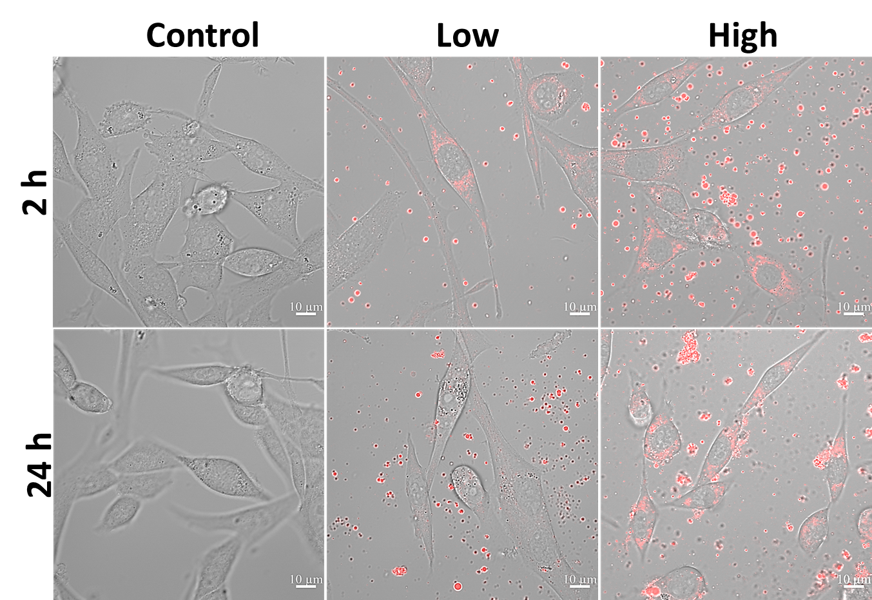


Figure S. 4. The internalization of fluorescent PLGA nanoparticles by E0771 cells is influenced by exposure time and particle concentration. Confocal images of E0771 cells exposed to low (16.5 µg/mL) and high (66.5 µg/mL) concentrations of PLGA-Cy5 fluorescent nanoparticles after 2 h and 24 h of incubation with the nanoparticles.


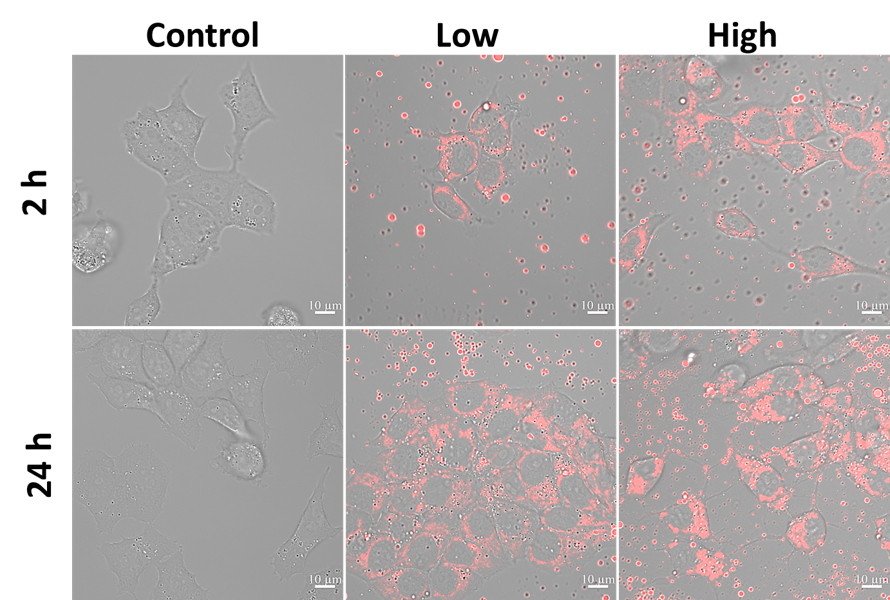


Figure S. 5. The internalization of fluorescent PLGA nanoparticles by MM231 cells is influenced by exposure time and particle concentration. Confocal images of MM231 cells exposed to low (16.5 µg/mL) and high (66.5 µg/mL) concentrations of PLGA-Cy5 fluorescent nanoparticles after 2 h and 24 h of incubation with the nanoparticles.


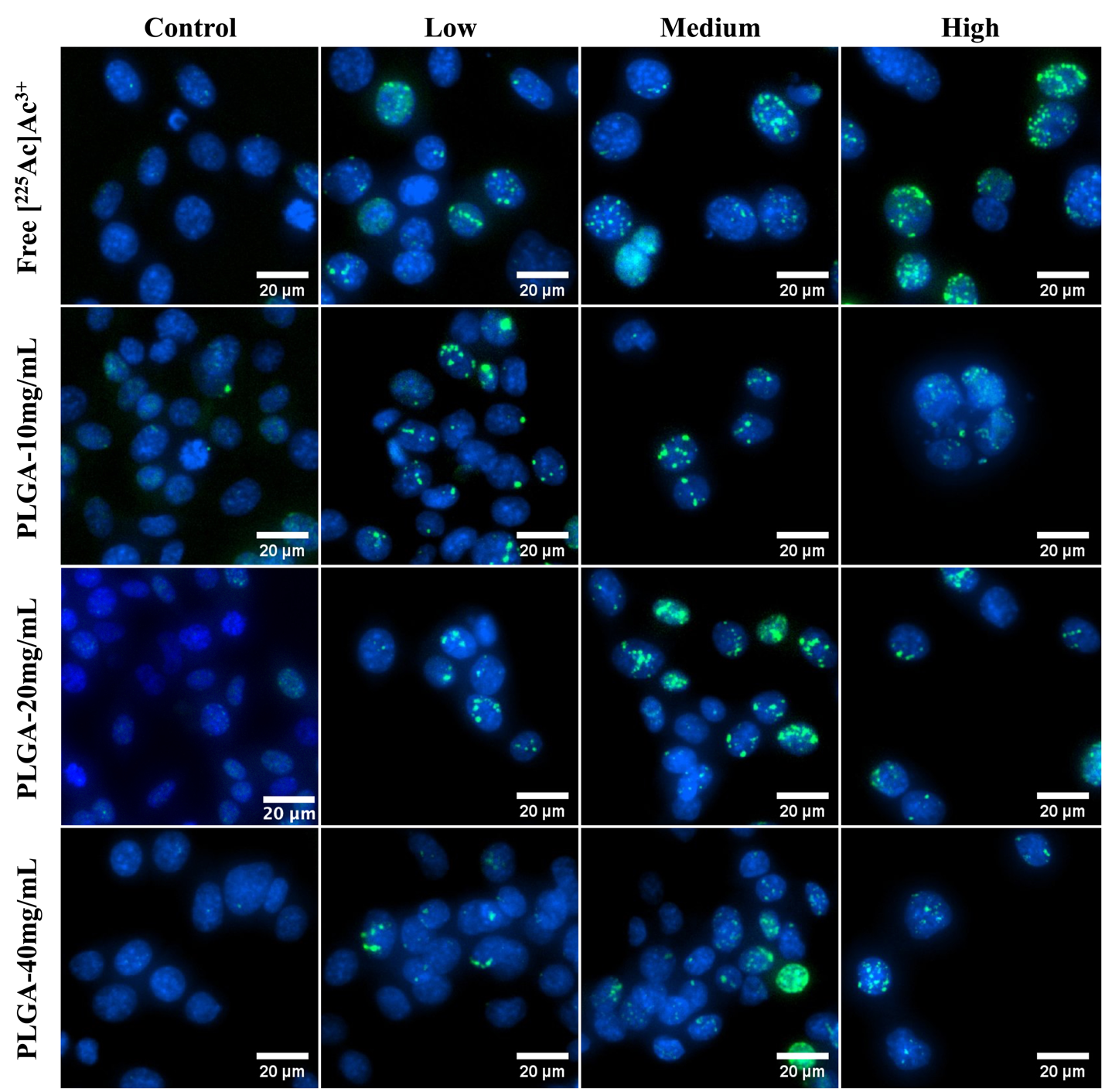


Figure S. 6 Alpha particles from ^225^Ac and its decay daughters induced the expression of γ-H2AX foci, indicative of DNA double strand breaks that can lead to cell death. Representative images of γ-H2AX foci in E0771 cells exposed to free [^225^Ac]Ac^3+^ (Low – 0.6 kBq/mL, Medium – 1.1 kBq/mL, High – 2.3 kBq/mL), PLGA([^225^Ac]AcBLPhen) [10 mg/mL] (Low – 0.4 kBq/mL, Medium – 0.8 kBq/mL, High – 1.7 kBq/mL), PLGA([^225^Ac]AcBLPhen) [20 mg/mL] (Low – 0.4 kBq/mL, Medium – 0.8 kBq/mL, High – 1.7 kBq/mL), and PLGA([^225^Ac]AcBLPhen) [40 mg/mL] (Low – 0.5 kBq/mL, Medium – 0.9 kBq/mL, High – 1.8 kBq/mL) for 24 h. γ-H2AX (green) and nuclear DNA stained with hoechst (blue). Scale bar is 20 µm and images were acquired using Cytation 1 Imager and Plate reader.


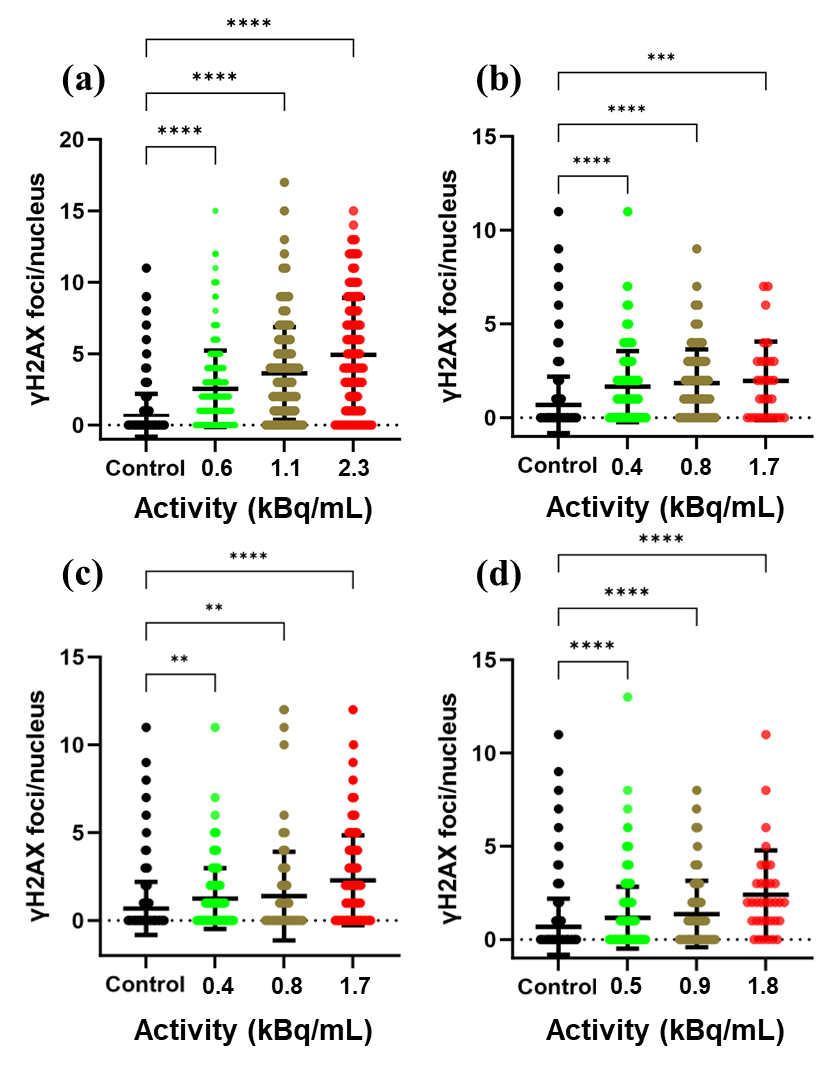


Figure S. 7. Increase of γ-H2AX foci indicating DNA double-strand breaks with higher ^225^Ac activities. γ-H2AX foci of DNA double-strand breaks quantified by an automated spot counting algorithm. Data summarizing analysis and quantification of individual γ-H2AX foci per E0771 nucleus after 24 h exposure to (a) free [^225^Ac]Ac^3+^ and PLGA nanoparticles encapsulating [^225^Ac]AcBLPhen at (b) 10 mg/mL, (c) 20 mg/mL, and (d) 40 mg/mL. Reported values correspond to the mean of 4 technical replicates and n = 1 experiment. **P*<0.05*,* ***P*<0.01, ****P*<0.001, *****P*<0.0001, one-way ANOVA followed by Tukey multiple comparisons post-test.


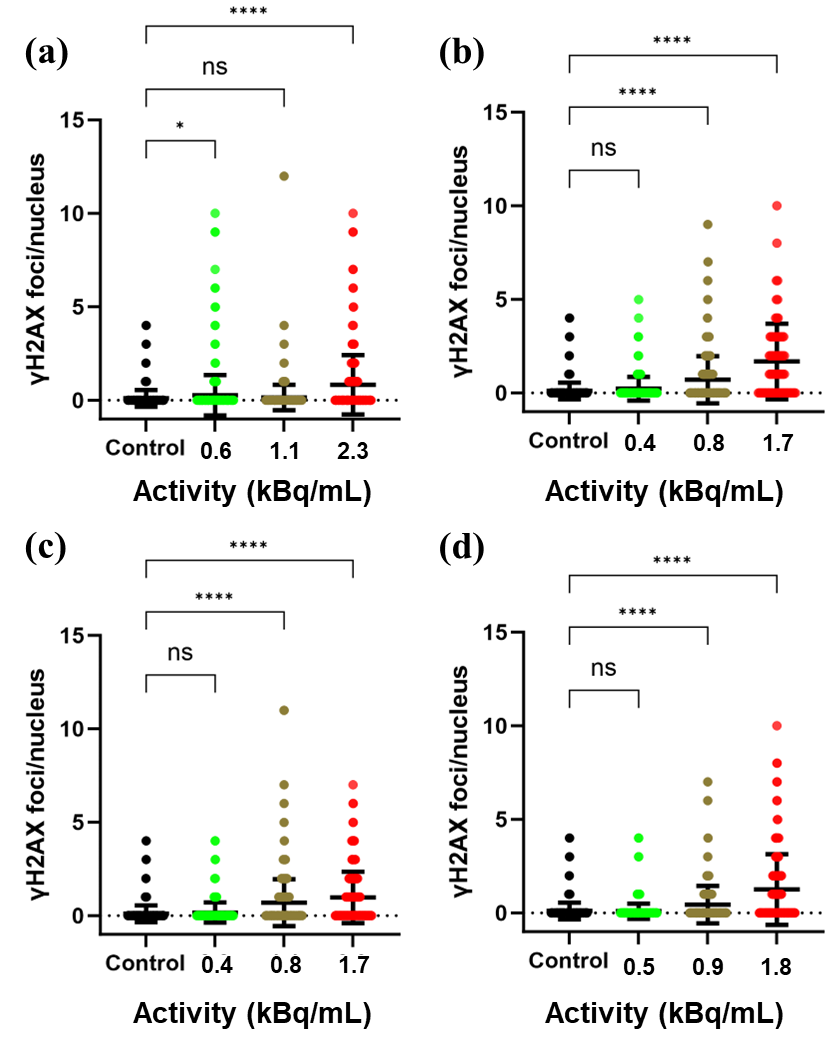


Figure S. 8. Increase of γ-H2AX foci indicating DNA double-strand breaks with higher ^225^Ac activities. γ-H2AX foci of DNA double-strand breaks quantified by automated spot counting algorithm. Data summarizing analysis and quantification of individual γ-H2AX foci per MCF7 nucleus after 24 h exposure to (a) free [^225^Ac]Ac^3+^ and PLGA nanoparticles encapsulating [^225^Ac]AcBLPhen at (b) 10 mg/mL, (c) 20 mg/mL, and (d) 40 mg/mL. Reported values correspond to the mean of 4 technical replicates and n = 1 experiment. **P*<0.05*,* ***P*<0.01, ****P*<0.001, *****P*<0.0001, one-way ANOVA followed by Tukey multiple comparisons post-test.


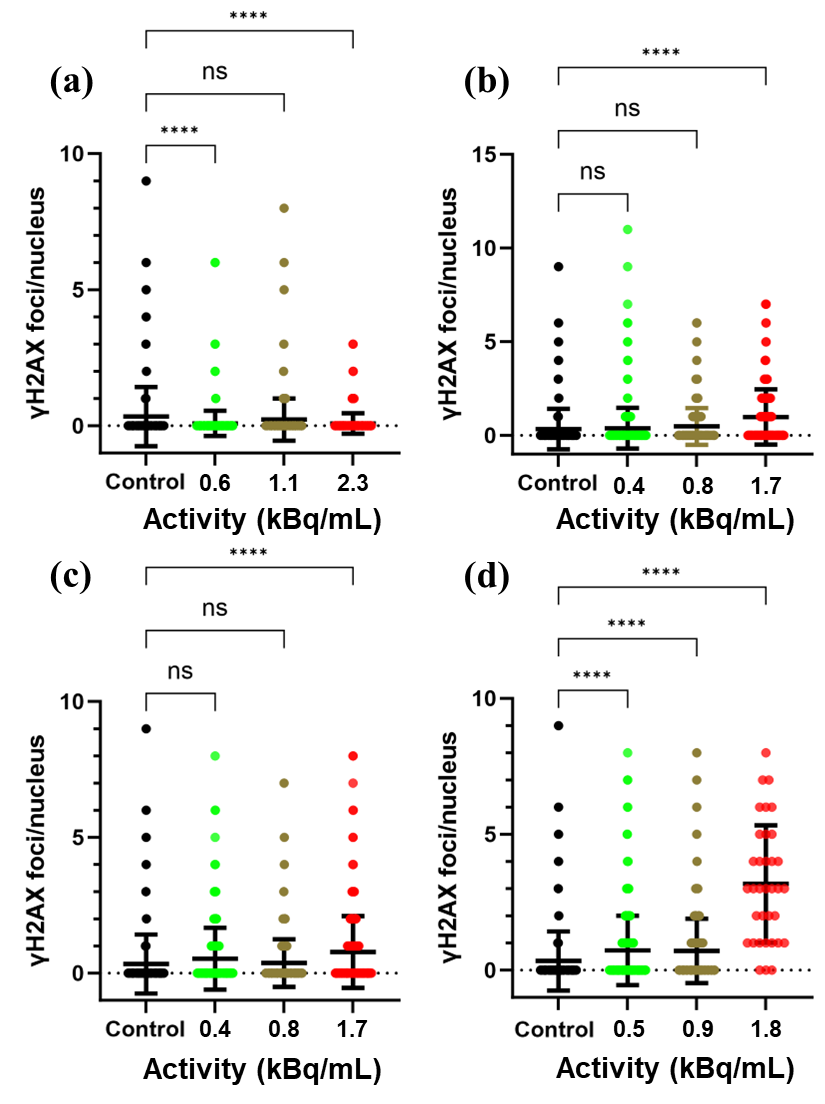


Figure S. 9. Increase of γ-H2AX foci indicating DNA double-strand breaks with higher ^225^Ac activities. γ-H2AX foci of DNA double-strand breaks quantified by automated spot counting algorithm. Data summarizing analysis and quantification of individual γ-H2AX foci per MM231 nucleus after 24 h exposure to (a) free [^225^Ac]Ac^3+^ and PLGA nanoparticles encapsulating [^225^Ac]AcBLPhen at (b) 10 mg/mL, (c) 20 mg/mL, and (d) 40 mg/mL. Reported values correspond to the mean of 4 technical replicates and n = 1 experiment. **P*<0.05*,* ***P*<0.01, ****P*<0.001, *****P*<0.0001, one-way ANOVA followed by Tukey multiple comparisons post-test.





Figure S. 10. Exposure time influences the cytotoxic effect of ^225^Ac delivered as free radionuclide and encapsulated within PLGA nanoparticles. Increasing ^225^Ac exposure time decreases E0771 cell viability for both free [^225^Ac]Ac^3+^ and [^225^Ac]AcBLPhen encapsulated within PLGA nanoparticles. Viability relative to untreated cells was evaluated (a) 24 h and (b) 48 h post-exposure to free ^225^Ac for different times. Similarly, viability relative to untreated cells was assessed (c) 24 h and (d) 48 h post-exposure to free [^225^Ac]Ac^3+^ and [^225^Ac]AcBLPhen encapsulated within PLGA nanoparticles for different times. An alamarBlue assay was used to evaluate cell viability; the reported values correspond to the mean of five technical replicates and n = 1 experiment. Error bars show the standard error of the mean.





Figure S. 11. Exposure time influences the cytotoxic effect of ^225^Ac delivered as free radionuclide and encapsulated within PLGA nanoparticles. Increasing ^225^Ac exposure time decreases MCF7 cell viability for both free ^225^Ac and ^225^Ac-BLPhen encapsulated within PLGA nanoparticles. Viability relative to untreated cells was evaluated (a) 24 h and (b) 48 h post-exposure to free ^225^Ac for different times. Similarly, viability relative to untreated cells was assessed (c) 24 h and (d) 48 h post-exposure to free [^225^Ac]Ac^3+^ and [^225^Ac]AcBLPhen encapsulated within PLGA nanoparticles for different times. An alamarBlue assay was used to evaluate cell viability; the reported values correspond to the mean of five technical replicates and n = 1 experiment. Error bars show the standard error of the mean.
